# Immunization with inactivated whole virus particle influenza virus vaccines improves the humoral response landscape in cynomolgus macaques

**DOI:** 10.1371/journal.ppat.1010891

**Published:** 2022-10-07

**Authors:** Brendon Y. Chua, Toshiki Sekiya, Marios Koutsakos, Naoki Nomura, Louise C. Rowntree, Thi H. O. Nguyen, Hayley A. McQuilten, Marumi Ohno, Yuki Ohara, Tomohiro Nishimura, Masafumi Endo, Yasushi Itoh, Jennifer R. Habel, Kevin J. Selva, Adam K. Wheatley, Bruce D. Wines, P. Mark Hogarth, Stephen J. Kent, Amy W. Chung, David C. Jackson, Lorena E. Brown, Masashi Shingai, Katherine Kedzierska, Hiroshi Kida

**Affiliations:** 1 Global Station for Zoonosis Control, Global Institution for Collaborative Research and Education (GI-CoRE) Hokkaido University, Sapporo, Japan; 2 Department of Microbiology and Immunology, The University of Melbourne at the Peter Doherty Institute for Infection and Immunity, Melbourne, Australia; 3 International Institute for Zoonosis Control, Hokkaido University, Sapporo, Japan; 4 KM Biologics Co. Ltd., Kumamoto, Japan; 5 Division of Pathogenesis and Disease Regulation, Department of Pathology, Shiga University of Medical Science, Otsu, Japan; 6 Immune Therapies Group, Burnet Institute, Melbourne, Australia; 7 Department of Immunology and Pathology, Central Clinical School, Monash University, Melbourne, Australia; 8 Department of Pathology, The University of Melbourne, Parkville, Australia; 9 Melbourne Sexual Health Centre, Infectious Diseases Department, Alfred Health, Central Clinical School, Monash University, Melbourne, Australia; Johns Hopkins Bloomberg School of Public Health, UNITED STATES

## Abstract

Although antibody-inducing split virus vaccines (SV) are currently the most effective way to combat seasonal influenza, their efficacy can be modest, especially in immunologically-naïve individuals. We investigated immune responses towards inactivated whole influenza virus particle vaccine (WPV) formulations, predicated to be more immunogenic, in a non-human primate model, as an important step towards clinical testing in humans. Comprehensive analyses were used to capture 46 immune parameters to profile how WPV-induced responses differed to those elicited by antigenically-similar SV formulations. Naïve cynomolgus macaques vaccinated with either monovalent or quadrivalent WPV consistently induced stronger antibody responses and hemagglutination inhibition (HI) antibody titres against vaccine-matched viruses compared to SV formulations, while acute reactogenic effects were similar. Responses in WPV-primed animals were further increased by boosting with the same formulation, conversely to modest responses after priming and boosting with SV. 28-parameter multiplex bead array defined key antibody features and showed that while both WPV and SV induced elevated IgG responses against A/H1N1 nucleoprotein, only WPV increased IgG responses against A/H1N1 hemagglutinin (HA) and HA-Stem, and higher IgA responses to A/H1N1-HA after each vaccine dose. Antibodies to A/H1N1-HA and HA-Stem that could engage FcγR2a and FcγR3a were also present at higher levels after one dose of WPV compared to SV and remained elevated after the second dose. Furthermore, WPV-enhanced antibody responses were associated with higher frequencies of HA-specific B-cells and IFN-γ-producing CD4^+^ T-cell responses. Our data additionally demonstrate stronger boosting of HI titres by WPV following prior infection and support WPV administered as a priming dose irrespective of the follow up vaccine for the second dose. Our findings thus show that compared to SV vaccination, WPV-induced humoral responses are significantly increased in scope and magnitude, advocating WPV vaccination regimens for priming immunologically-naïve individuals and also in the event of a pandemic outbreak.

## Author summary

Influenza viral infections cause significant morbidity and mortality. Although current split virus (SV) vaccines are the most effective way to combat seasonal influenza, their efficacy can be modest. We investigated immune responses directed towards inactivated whole influenza virus particle vaccine (WPV) formulations in a non-human primate model as an important step towards clinical testing in humans. Our analysis of 46 immune parameters found that compared to SV vaccination, WPV-induced antibody responses were significantly increased in scope and magnitude, together with higher frequencies of influenza-specific B-cell and CD4^+^ T-cell responses. Our study provides key insights into the immune responses induced by WPV vaccination in immunologically-naïve animals.

## Introduction

A/H1N1 and A/H3N2 influenza A viruses (IAV), together with influenza B viruses (IBV), are responsible for the majority of seasonal influenza outbreaks in the human population [1]. As a result of global travel restrictions and social measures to curb the impact of the COVID-19 pandemic, however, influenza cases and deaths around the world have since dropped to unprecedented levels [2]. However, as countries now begin open their borders, without the natural boost to immunity that seasonal influenza would have provided in the past 18 months, leaves populations vulnerable to severe influenza seasons. Therefore, important pre-emptive measures to counteract potentially serious influenza outbreaks need to be in place. In Japan, for example, levels of protective antibodies (as defined by hemagglutination inhibition titers prior to seasonal vaccination) against IAV and IBV strains have decreased substantially especially in the young and elderly since the advent of the pandemic [3, 4]. In countries such as Australia, higher than average rises in infection cases have been reported as measures to curb transmission are lifted [5], despite increases in seasonal influenza vaccination rates [6].

Influenza vaccination that induces neutralizing antibodies against viral hemagglutinin (HA) and neuraminidase (NA) surface glycoproteins is the most effective way to control the impact of seasonal influenza. However, despite this, up to 5 million cases of severe infections and 243,000 to 640,000 deaths still occurred worldwide annually in pre-COVID-19 years [7]. The most commonly used influenza vaccines comprise of ether- or detergent-disrupted “split” virus vaccines (SV), available as trivalent or quadrivalent formulations to target two IAV and one or two IBV strains. Their effectiveness largely depends on how well-matched the vaccine strains are with the strains that ultimately come to circulate in the upcoming influenza seasons. As a result of continual antigenic drift in the HA, appropriate viral strains to use in future years are not always available or not predicted with certainty, leading to greatly reduced vaccine effectiveness in some seasons or there can potentially be a strain mismatch [1,8,9]. Strains included in influenza vaccines are therefore scrutinized annually and updated when required to maintain protection in the population. Numerous studies have shown that SV vaccination remains suboptimal at inducing and/or boosting immunity in previously primed populations, including the elderly and immunocompromised individuals, and can be less effective in naïve populations such as young children [10, 11]. Furthermore, reports of declining vaccine effectiveness over several years [12, 13] are a cause for concern, especially in the event of severe seasonal influenza epidemics. There is therefore an ongoing need to develop more effective and safe vaccine strategies.

First introduced in the 1940’s, vaccination with inactivated whole influenza virus particle vaccines (WPV), although effective at conferring protection, were often associated with local and systemic adverse effects typified by excessive inflammatory responses and fever [14–16]. Concerns of their reactogenicity have been largely alleviated by improvements in techniques to remove impurities [17], the production of virus stocks in cell culture-based systems [18] as well as a better understanding of the immune-mediated mechanisms that underpin their immunogenicity [19, 20]. Several studies have since revisited the use of WPV formulations and demonstrated that they are well-tolerated and immunogenic in adult and elderly subjects [21–23] as well as children and adolescents [24].

The All-Japan Influenza Vaccine Study Group was established to develop safe and immunogenic WPVs for use in humans, with the involvement of all four of Japan’s seasonal influenza vaccine manufacturers. WPV formulations prepared by each manufacturer has been shown to induce stronger neutralizing antibodies and innate immune responses in mice compared to SV formulations consisting of the same vaccine strains. To predict the potential efficacy of these WPV formulations in humans before transitioning to clinical trials requires evaluations in animal models that bear close genetic and physiological similarities to humans versus other species such as rodents. In this regard, non-human primates are ideal as their immune responses elicited by infection or vaccination can closely reflect those in humans. However, the ability to perform in-depth analysis of the immune mechanisms that underpin the generation of protective immunity in non-human primates, particularly against influenza, is limited by the availability of reagents and assays suited to this task. We have recently established detection methods for analysing and understanding immune responses in influenza virus-infected cynomolgus macaques [25]. In this study, we applied these techniques combined with a comprehensive suite of additional analytical methods capturing 46 immune parameters, to investigate the immunogenicity of good manufacturing practice (GMP) grade monovalent and quadrivalent WPV and SV formulations comprising the 2018–2019 Japanese influenza seasonal strains in cynomolgus macaques. Our in-depth analyses of antibody responses (their isotypes/capacity to engage FcγR binding against a range of influenza virus antigens and inhibition of hemagglutinin), together with cytokine responses, antigen-specific B cell and cytokine-producing T cell responses provide an extensive dataset on how vaccination of naïve animals with WPV formulations consistently induce stronger humoral responses against vaccine-matched virus strains as well as enhance responses in those previously infected with influenza viruses.

## Results

### Monovalent WPV induces stronger protective antibody responses than SV, with similar reactogenicity

**Fig 1:**
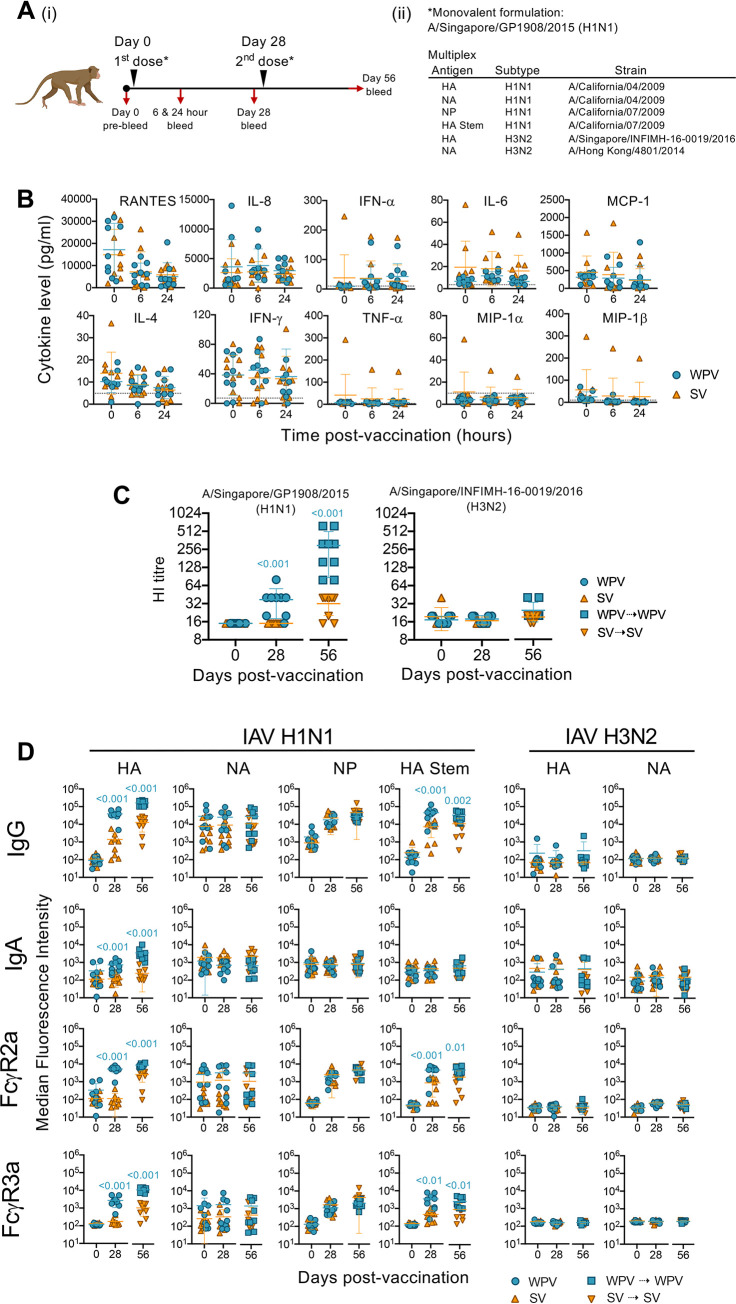
Monovalent WPV induces stronger antibody and HI responses than SV. (A[i]) Macaques (n = 9 per group) were vaccinated with monovalent A/Singapore/GP1908/2015 (H1N1) WPV or SV followed by a second dose 28 days later. Blood was collected prior to receiving each dose (day 0 pre-bleed and day 28) and at day 56. (A[ii]) List of antigen and strain information used for multiplex analysis. (B) Cytokine levels in plasma obtained at 0, 6 and 24 hours after the first dose. Dotted lines indicate the manufacturer’s theoretical limit of detection. (C) Hemagglutination inhibition (HI) was measured against A/Singapore/GP1908/2015 (H1N1) and A/Singapore/INFIMH-16-0019/2016 (H3N2). Individual HI antibody titres are expressed as reciprocals of the highest plasma dilutions that showed complete inhibition of agglutination. (D) Multiplex analysis of antibody responses in plasma samples against H1N1 and H3N2 antigens. Median fluorescence intensity of IgG, IgA, FcγR2a and FcγR3a binding to HA, NA, NP and HA Stem from H1N1 strains as well as HA and NA from H3N2 strains. Mann-Whitney tests were used to analyse statistical significance between vaccine groups at each time point with the mean±SD in each group shown. Figure created with Biorender.

We first compared the immunogenicity of monovalent WPV and SV A/Singapore/GP1908/2015 (H1N1) formulations containing equivalent amounts of HA by vaccinating naïve cynomolgus macaques via the sub-cutaneous route (Fig 1A[i]), the most prevalent vaccination route used for humans in Japan. Each animal (n = 9 per vaccine type) received two vaccine doses and was bled for the assessment of antibody prior to the first (day 0) and second dose (day 28) as well as 28 days after the second dose (day 56). Blood was also obtained at 6 and 24 hours after the first dose to define inflammatory cytokine/chemokine responses. At both these time points, levels of RANTES, IL-8, IFN-α, IL-6, MCP-1, IL-4, IFN-γ, TNF-α, MIP-1α and MIP-1β remained similar following SV and WPV vaccination relative to baseline levels (Fig 1B), indicating that inflammatory responses, at least in blood, induced by WPV and SV inoculation were minimal. Furthermore, there were no differences in body temperature, weight and food intake exhibited by animals in each group (S1 Fig) nor were there consistent clinical signs and injection site reactions across each group (S1 Table).

To determine antibody responses following immunization with WPV and SV, we firstly performed hemagglutination-inhibition (HI) assays. Significantly higher A/H1N1-specific HI antibody titres were induced by WPV after both first and second doses (2.5-fold and 9.4-fold, respectively) against the vaccine-matched virus strain (P<0.001; Fig 1C) in comparison to HI antibody titres elicited by SV. Increases in HI antibody titres were not observed against a heterologous strain, A/Singapore/INFIMH-16-0019/2016 (H3N2), in all vaccine groups.

To simultaneously define multidimensional antibody features elicited after WPV and SV immunization, a 28-parameter multiplex bead array was performed [26, 27]. Trimeric HA and monomeric NA (from A/H1N1, A/H3N2 and B/Phuket) as well as HA Stem (from A/H1N1) and NP antigens were used for detecting a range of epitope-specific antibody isotypes (IgA and IgG) and Fcγ receptor binding (FcγR2a and FcγR3a) (Fig 1A[ii] and S2 Table). After one vaccine dose, both WPV and SV induced elevated IgG responses against A/H1N1 NP (WPV; P = 0.014 and SV; P = 0.007 on day 28 compared to day 0; S2A Fig), but only WPV induced significantly increased IgG responses against A/H1N1 HA (P = 0.048) and HA Stem (P<0.001) which were further boosted after the second dose (P≤0.001). Importantly, when compared to SV, WPV-vaccination induced significantly higher IgG levels against A/H1N1 HA (P<0.001 on day 28 and 56) and HA Stem (P<0.001 on day 28 and P = 0.002 on day 56) after each vaccine dose (Fig 1D). Higher A/H1N1 HA (P = 0.002 on day 28 and P<0.0001 on day 56) and HA Stem-specific antibodies (P = 0.0019 on day 28 and 0.0106 on day 56) in WPV-vaccinated animals were also validated by ELISA (S3A Fig).

Higher IgA responses to A/H1N1 HA (P<0.001) were also induced after each dose of WPV compared to SV. When FcγRs were examined across different antigen specificities, one WPV dose was sufficient to induce significantly higher levels of FcγR2a and FcγR3a-binding antibodies to A/H1N1 HA (both P<0.001) and HA Stem (P<0.001 and P<0.01, respectively) compared to an equivalent dose of SV, and remained higher after the second dose. The increased binding to FcγR2a and FcγR3a soluble dimers for HA and HA Stem-specific antibodies suggests that, in addition to their potential ability to neutralize virus infectivity, they can facilitate antibody-mediated activity such as antibody-dependent cellular cytotoxicity (ADCC) and antibody-dependent cellular phagocytosis (ADCP). As these were vaccine monovalent formulations, vaccination with either vaccine type did not induce responses against A/H3N2 HA and NA.

**Fig 2:**
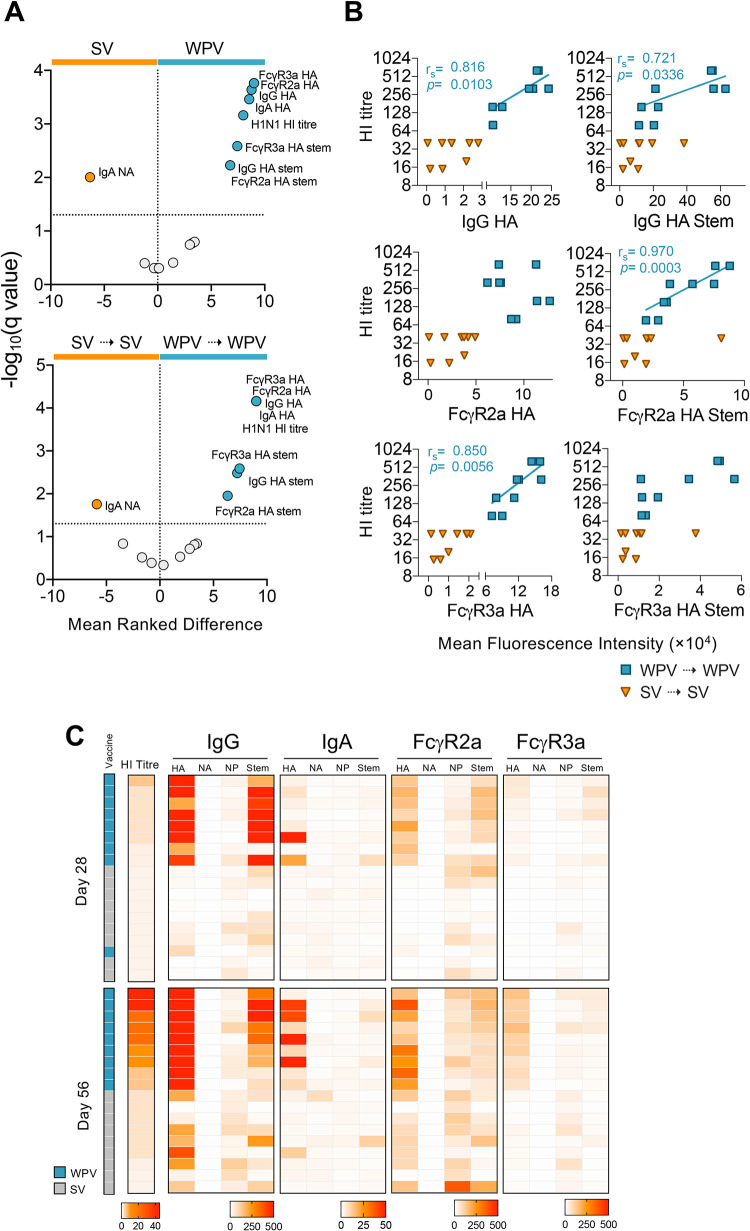
HI titres induced by WPV strongly correlate with IgG responses and FcγR usage against H1N1 antigens. (A) Volcano plots of 17 humoral immune parameters following either one (SV or WPV) or two doses (SV⇢SV or WPV⇢WPV) of monovalent vaccine are shown. Mean ranked differences and q values were determined using Mann-Whitney tests of Log-transformed data, with a false discovery rate <5% considered statistically significant. (B) Correlation between A/H1N1 HI antibody titres and IgG, FcγR2a and FcγR3a against A/H1N1 HA and HA Stem (on day 56) in macaques vaccinated with 2 doses of WPV or SV. Spearman’s ranked correlation coefficients (r_s_) are depicted only for paired comparisons with significant positive correlations (P<0.05). (C) Heatmap of A/H1N1-specific HI antibody titres and antibody levels in macaques after vaccination with one (day 28) or two doses (day 56) of monovalent WPV or SV. Each row represents a different macaque with their matched measurements in each column.

Analysis of 17 humoral immune parameters revealed key antibody features that were significantly elevated for WPV in comparison to SV, after both single or double dose immunization, including FcγR3a HA, FcγR2a HA, IgG HA, IgA HA, IgG HA stem, FcγR3a HA stem, FcγR2a HA stem and H1N1 HI antibody titres (Fig 2A). Having observed strong antibody responses elicited by WPV, we further investigated correlates of strong HI antibody titres in the context of key antibody and FcγR features detected by the multiplex bead array (Fig 2B). We found that A/H1N1 HI antibody titres positively correlated with IgG antibodies specific for A/H1N1 HA (r_s_ = 0.816) and HA Stem (r_s_ = 0.721) in WPV-vaccinated macaques after two vaccine doses (day 56). Interestingly, when we examined correlations with FcγR usage, HI titres were associated with FcγR2a-binding responses of HA Stem-specific antibodies (r_s_ = 0.970) as well as FcγR3a-binding responses to HA-specific antibodies (r_s_ = 0.850), again suggesting their potential to facilitate multiple mechanisms of antibody-mediated protection.

To further analyze the magnitude of antibody responses in each macaque, we collated a heatmap based on the fold-change in HI antibody titres and A/H1N1 antigen-specific antibody responses after the first and second doses (on day 28 and day 56 respectively), relative to their baseline levels (day 0; Fig 2C). When we ranked animals according to their HI antibody titres, the strongest increases in antibody responses were observed after each WPV dose. The most prominent increases were for IgG antibodies as well as FcγR2a- and FcγR3a-binding responses against HA and HA Stem, in line with the correlations described above. While strong IgA responses against HA were also observed in some WPV-vaccinated animals, this was not proportional to HI antibody titre levels. Overall, our data provide evidence for increased antibody responses elicited by monovalent WPV in comparison to SV immunization, while reactogenicity towards both vaccines was similar.

### Quadrivalent WPV formulation induces strong antibody responses associated with increased antigen-specific B cell frequencies

**Fig 3:**
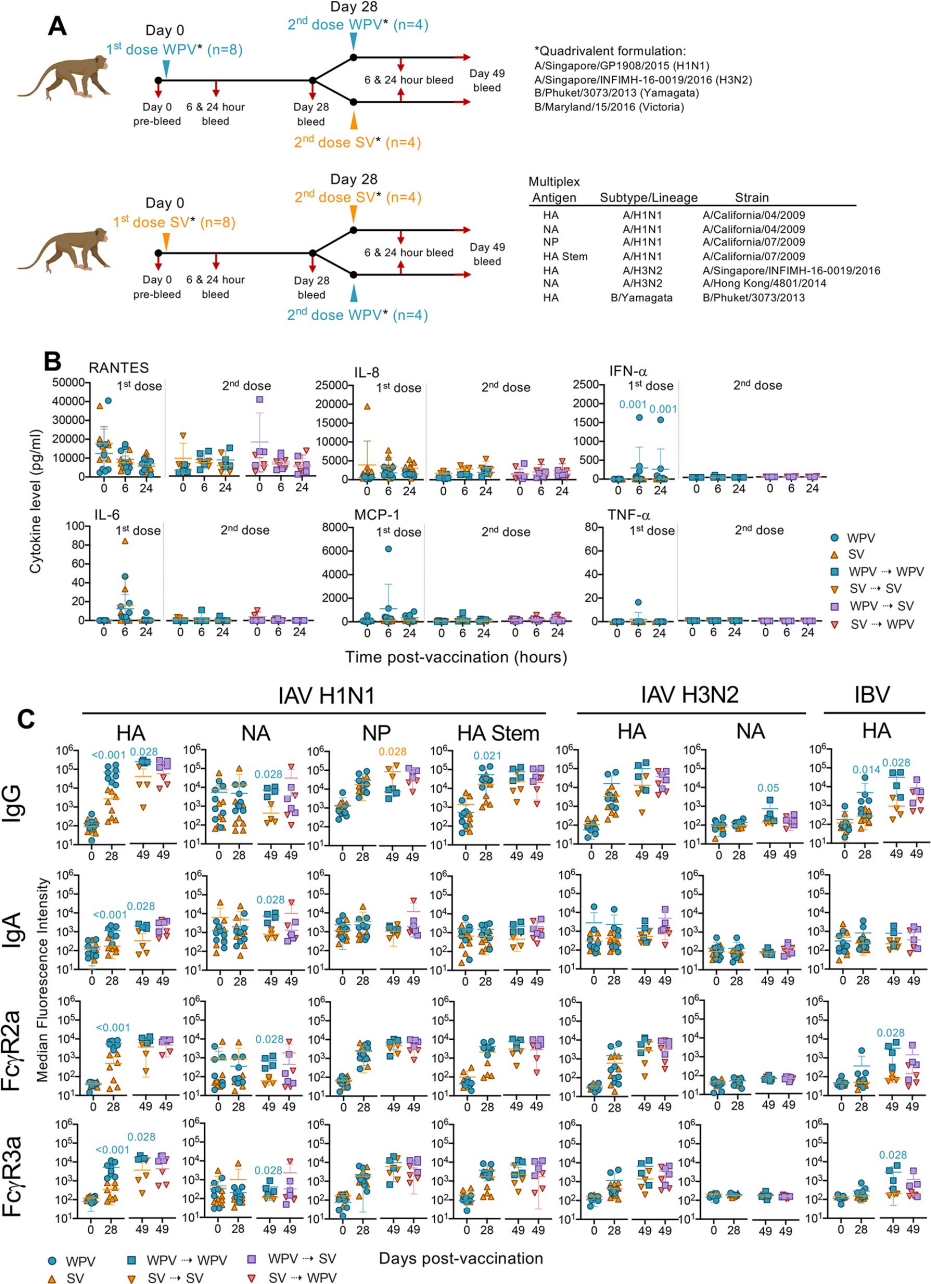
Quadrivalent WPV induces stronger antibody responses to vaccine matched strains than SV. (A) Macaques (n = 8 per group) were vaccinated with quadrivalent WPV or SV formulations. A second dose of either the same (n = 4) or opposite formulation to the first vaccine used (n = 4) was administered 28 days later. (B) Cytokine levels in the plasma obtained at 6 and 24 hours following each dose compared to their baseline levels at day 0. Dotted lines indicate the manufacturer’s theoretical limit of detection. (C) Multiplex analysis of antibody responses against A/H1N1, A/H3N2 and IBV antigens. Mann-Whitney tests were used to analyze statistical significance between vaccine groups at each time point with the mean±SD shown. Figure created with Biorender.

Having shown strong antibody responses elicited by monovalent WPV, we subsequently compared immune responses induced by quadrivalent WPV and SV containing A/H1N1 (A/Singapore/GP1908/2015), A/H3N2 (A/Singapore/INFIMH-16-0019/2016), B/Yamagata (B/Phuket/3073/2013) and B/Victoria (B/Maryland/15/2016) strains. Macaques were first inoculated with either vaccine on day 0 (n = 8 per vaccine type). On day 28, half of the animals from each group (n = 4) were immunized with the same vaccine used for the first dose, while the remaining animals received a mixed vaccine regimen (Fig 3A[i]).

Increased IFN-α levels were detected after the first WPV dose compared to SV (P = 0.001 at 6 and 24 hours) (Fig 3B) which was not observed using the monovalent WPV and could be due to higher HA content in the quadrivalent formulation. No significant increases in RANTES, IL-8, IL-6, MCP-1 and TNF-α after one or two doses were observed, irrespective of the vaccine regimen used.

Significant increases in antibody responses were observed more so after a single dose of WPV than SV (on day 28 compared to day 0) across different antigen specificities (S2B Fig). This translated to significantly higher (P<0.001) levels of IgG, IgA, FcγR2a- and FcγR3a-binding responses of A/H1N1 HA-specific antibodies, as well as IgG to HA Stem (P = 0.021) and B/Yamagata HA (P = 0.014) induced by WPV compared to SV (Fig 3C). These responses, to a large extent, remained elevated after a second WPV dose (WPV⇢WPV; on day 49) and were still significantly higher (IgG, IgA, FcγR3a-expressing antibodies to A/H1N1 HA, P = 0.028; IgG to IBV HA, FcγR2a expressing antibodies to IBV HA; P = 0.028) than when an SV regimen was used (SV⇢SV; on day 49). However, responses towards A/H3N2 antigens induced by both vaccine regimens were equivalent.

In animals that received the mixed vaccine regimens, there was a consistent trend in animals vaccinated with WPV followed by SV (WPV⇢SV) to exhibit higher responses (on day 49), particularly against A/H1N1 HA and HA Stem and the HA of B/Yamagata, in comparison to the opposite regimen (SV⇢WPV) (Fig 3C). Comparing these responses to those elicited with two doses of the same vaccine demonstrated that antibody levels induced by WPV⇢SV were of a similar magnitude to those induced by a double dose WPV⇢ WPV (S4A Fig). The WPV⇢SV regimen tended to induce significantly higher responses than a double dose of SV (SV⇢SV), particularly IgG, IgA and FcγR3a-expressing antibodies to A/H1N1 HA and IgG to IBV HA. In contrast, when WPV was given as a second dose to SV-vaccinated animals (SV⇢WPV), antibody levels remained similar to those found in animals that received the SV⇢SV regimen. This suggests advantages for WPV to be given as a priming dose irrespective of the follow up vaccine type used for the second dose.

**Fig 4:**
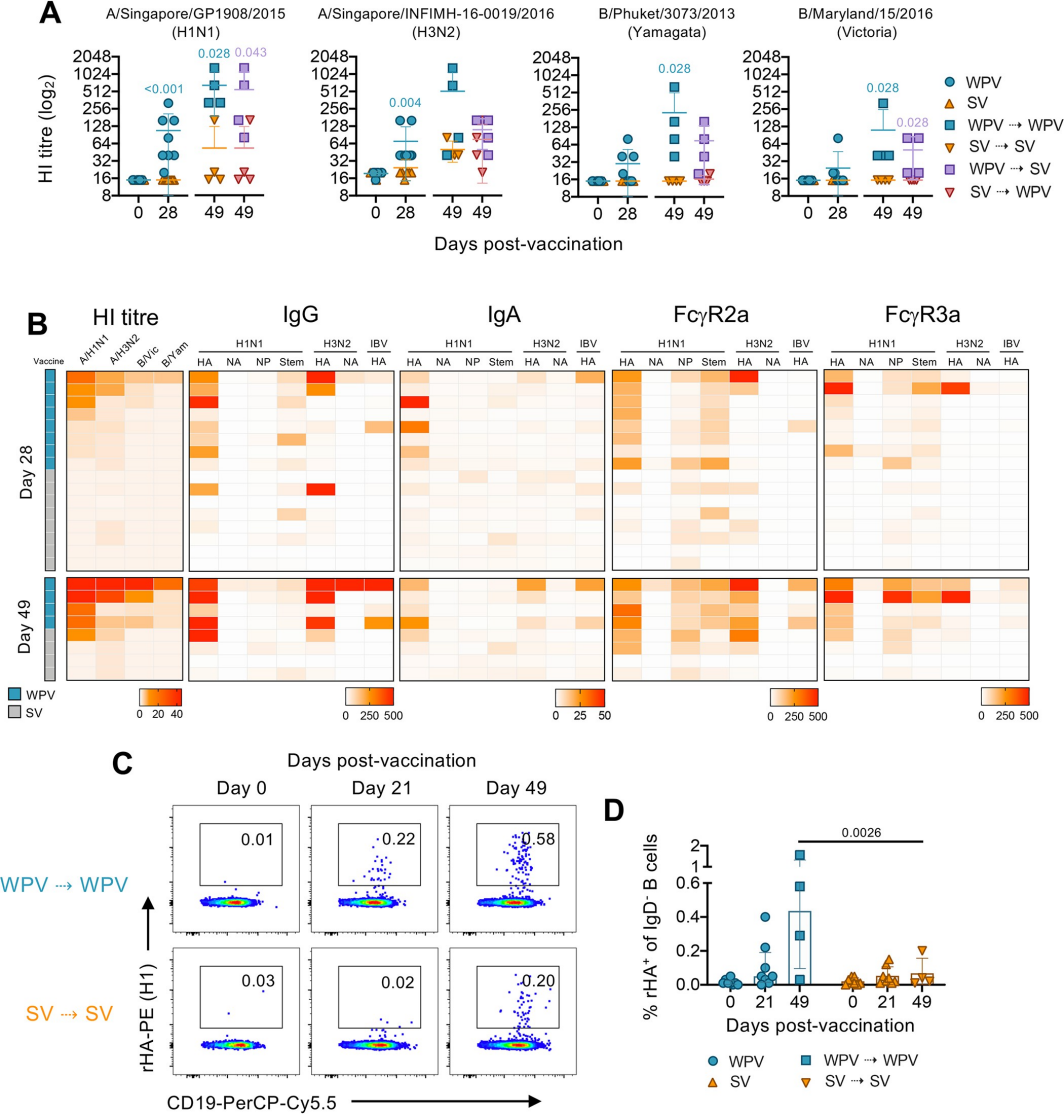
Increased HI titres induced by WPV are associated with superior antibody profiles and correlate with higher frequencies of antigen-specific B cells. (A) Plasma collected at each time point was assessed for HI against vaccine-matched H1N1, H3N2 and IBV strains. Individual HI antibody titres are depicted with the mean±SD. (B) Heatmap of HI antibody titres and antibody levels of macaques after vaccination with one (day 28) or two doses (day 49) of the same formulation. Each row represents a different sample with their matched measurements in each column. (C) PBMCs isolated from macaques that were vaccinated with the same formulations were analysed for H1N1pdm09 virus-specific B cells. Shown are representative dot plots of rHA^+^CD19^+^ cells within class switched (IgD^-^) B cells and (D) frequencies (%) of rHA^+^ B cells within each group (mean±SD). Statistical significance in (A) data comparing WPV and SV groups at each time point was assessed by Mann-Whitney tests and (D) data comparing both groups and time points with each other was determined by a two-way ANOVA with Tukey’s correction for multiple comparisons.

Increased antibody responses induced by WPV also translated into elevated HI antibody titres against each of the viral strains used in the formulation. Significantly higher titres were detected against A/H1N1 (P = <0.001) and A/H3N2 (P = 0.004) after one WPV dose compared to SV (Fig 4A). In fact, titres in WPV-vaccinated animals were boosted and remained higher following a second dose of the same vaccine (WPV⇢WPV), particularly against A/H1N1 (P = 0.028) and both IBV strains (P = 0.028) compared to SV-vaccinated animals. Furthermore, HI antibody titres against IBV strains in macaques primed with WPV and then SV (WPV⇢SV) were not only higher in comparison to the converse regimen (SV⇢WPV), but also in comparison to animals that received two doses of SV, with a similar trend observed against A/H1N1 (Figs 4A and S4B). Thus, similar to the results observed for monovalent WPV, a quadrivalent formulation also led to better antibody responses, compared to SV immunization, with respect to broader immunity against different strains when administered sequentially and beneficial when used as a priming dose in a mixed vaccine regimen.

A ranked comparison of antibody responses according to HI antibody titres against each strain revealed once again that double-dosed WPV vaccinated animals with the highest titres also had the strongest IgG levels as well as dimeric FcγR2a and FcγR3a binding antibodies, especially to the HA of A/H1N1 in comparison to SV-vaccinated animals (Fig 4B). Antibody responses against A/H3N2 HA and B/Yamagata HA were also prominent albeit less consistent. Of note, however, strong HI antibody titres were also associated with FcγR2a-, and to some extent, FcγR3a-expressing antibodies to A/H1N1 HA Stem, suggesting the involvement in cross-reactive responses.

We have recently established the use of recombinant antigen HA (rHA) probes to define antigen-specific class-switched IgD^-^ CD19^+^ B cells in cynomolgus macaques [25]. Using this approach, rHA^+^ B cells specific for A/H1N1 HA (A/California/07/2009) (S5A Fig) could be readily detected in vaccinated animals after one dose of either vaccine (WPV; mean 0.11%, SV; mean 0.05%), with significantly higher frequencies (P = 0.0026) observed in animals that received two doses of WPV (mean 0.62%) in comparison to SV immunisation per se (mean 0.07%) (Fig 4C and 4D).

### WPV boosts immune responses in macaques previously infected with influenza viruses

**Fig 5:**
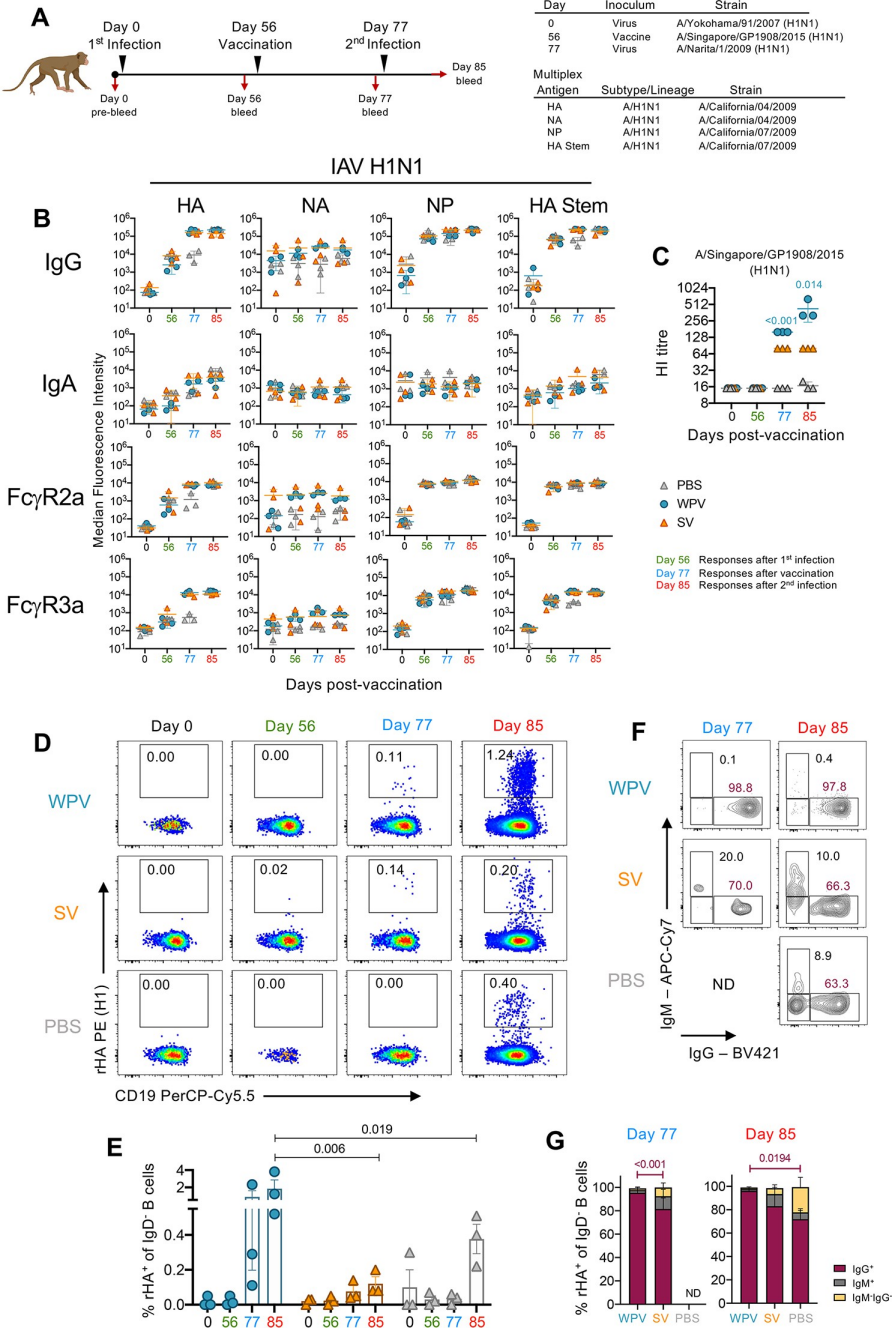
Vaccination boosts antibody responses in previously infected animals, while WPV induces stronger HI titres and B cell responses. (A) Macaques (n = 3 per group) were infected with A/Yokohama/91/2007 (H1N1) and 56 days later vaccinated with PBS or monovalent A/Singapore/GP1908/2015 (H1N1) WPV or SV. All macaques were then infected on day 77 with A/Narita/1/2009 (H1N1). Blood samples were collected prior to the first infection (day 0 pre-bleed), vaccination (day 56), second infection (day 77) and at the end of the experiment (day 85). (B) Multiplex analysis of antibody responses in plasma samples against H1N1 antigens (mean±SD). (C) Plasma was assessed for HI against A/Singapore/GP1908/2015 (H1N1) across the various time points (mean±SD). Detection of A/H1N1 virus-specific B cells in PBMCs showing (D) representative dot plots and (E) graphed data of class switched (IgD^-^) rHA^+^CD19^+^ cells and their frequencies (%) within each group (mean±SD). (F) Isotype distributions for rHA^+^ B cell populations in PBMCs on day 77 and 88. Representative FACS plots of IgG^+^ versus IgM^+^ B cells. (G) Bar graph indicating % isotype of rHA^+^ B cells. Indicated p-values reflect statistically significant differences in %rHA^+^IgG^+^ B cells between denoted groups. Analysis of (B, C) data comparing all groups at each time point was assessed by Kruskal-Wallis tests and (E, G) data comparing all groups and time points with each other was determined by two-way ANOVA with Tukey’s correction for multiple comparisons. Figure created with Biorender.

As much of the human population has pre-existing influenza-specific immunity, we next determined the immunogenicity of A/H1N1 WPV and SV immunization regimens in macaques previously infected with influenza virus to understand how pre-existing immunity affects vaccine responses. Macaques were first infected with the seasonal strain, A/Yokohama/91/2007 (H1N1), and subsequently vaccinated 56 days later with monovalent A/Singapore/GP/1908/2015 (H1N1) WPV or SV (Fig 5A). As controls, a subset of infected animals was inoculated with PBS only. We also assessed how immune responses are affected by a follow up influenza virus infection with the 2009 pandemic strain, A/Narita/1/2009 (H1N1), at 21 days after vaccination (day 77). We used these viruses as macques are known to be susceptible to infection with these strains [25, 28].

Comparable antibody responses induced by the first A/Yokohama/91/2007 (H1N1) infection (on day 56) were detected in all macaques prior to vaccination and typified by ~2log_10_-fold increases in IgG levels to A/H1N1 HA, NP and HA Stem with similar trends observed for FcγR2a and FcγR3a usage (Fig 5B). Vaccination with WPV and SV, but not PBS, led to increases in antibody responses (on day 77) particularly IgG (WPV, 78.4-fold, P = 0.023; SV, 19.2-fold, P = 0.011), FcγR2a- (WPV, 12.4-fold, P = 0.006; SV, 5.9-fold, P = 0.046) and FcγR3a- (WPV, 43.2-fold, P = 0.018; SV, 12.5-fold, P = 0.016) expressing antibodies to HA and IgG (WPV, 3.7-fold, P = 0.007; SV, 3.6-fold, P = 0.02) and FcγR3a-expressing antibodies (WPV, 3.4-fold, P = 0.008; SV, 2.8-fold, P = 0.042) to HA Stem. While these antibody responses increased (IgG HA, 26-fold, P<0.001; IgG HA Stem, 4.4-fold, P = 0.007; FcγR2a HA, 7.3-fold, P = 0.035; FcγR3a HA, 28.4-fold, P = 0.005; FcγR3a HA Stem, 4.7-fold, P = 0.005) in PBS-inoculated animals (on day 85) following the second influenza virus infection, responses did not increase in animals that received WPV and SV nor were there any differences between these two vaccine groups. A possible reason for this could be that these antibody levels were relatively high and beyond the detection limits of the array. We therefore performed an ELISA which showed that vaccination with WPV resulted in higher IgG HA- (day 77, P = 0.0103; day 85, P = 0.0013) and HA Stem- (day 77, P = 0.022) compared to vaccination with SV (S3B Fig). No differences in NP-specific titres were observed between both vaccine groups but PBS-inoculated animals exhibited higher responses at day 85 (P = 0.0011 compared to SV and P = 0.003 compared to WPV) which may be due to higher viral load in these animals.

As expected, initial infection with A/Yokohama/91/2007 did not elicit HI antibody titres on day 56 against the vaccine strain A/Singapore/GP/1908/2015 in all groups (Fig 5C). On day 77, however, WPV-induced HI antibody titres were significantly higher (2-fold, P<0.001) than those induced by SV and were further boosted by the second A/Narita/1/2009 influenza virus infection on day 85 (5.3-fold, P = 0.014). In line with this, we detected higher frequencies of rHA^+^ B cells in WPV-vaccinated animals (Fig 5D and 5E), particularly after the second influenza virus infection (WPV; mean 1.85%, SV; mean 0.12%, P = 0.006, PBS; mean 0.37%, P = 0.019). Furthermore, the majority of class-switched rHA^+^ B cells following vaccination with WPV were of IgG isotype where significantly higher proportions of IgG^+^rHA^+^ B cells were found compared to those induced by SV (day 77, WPV; mean 95.4%, SV; mean 81.6%, P<0.001)(Fig 5F and 5G). In contrast, higher frequency of IgM^+^ rHA^+^ B cells were found in animals immunised with SV compared to WPV (SV: mean 10.8%; WPV: mean 2.3%). Immune responses induced by both SV and WPV vaccination were sufficient for viral clearance as detectable viral titres were only observed in nasal swabs from PBS but not SV- and WPV-vaccinated animals following A/Narita/1/2009 infection (S5 Table).

**Fig 6:**
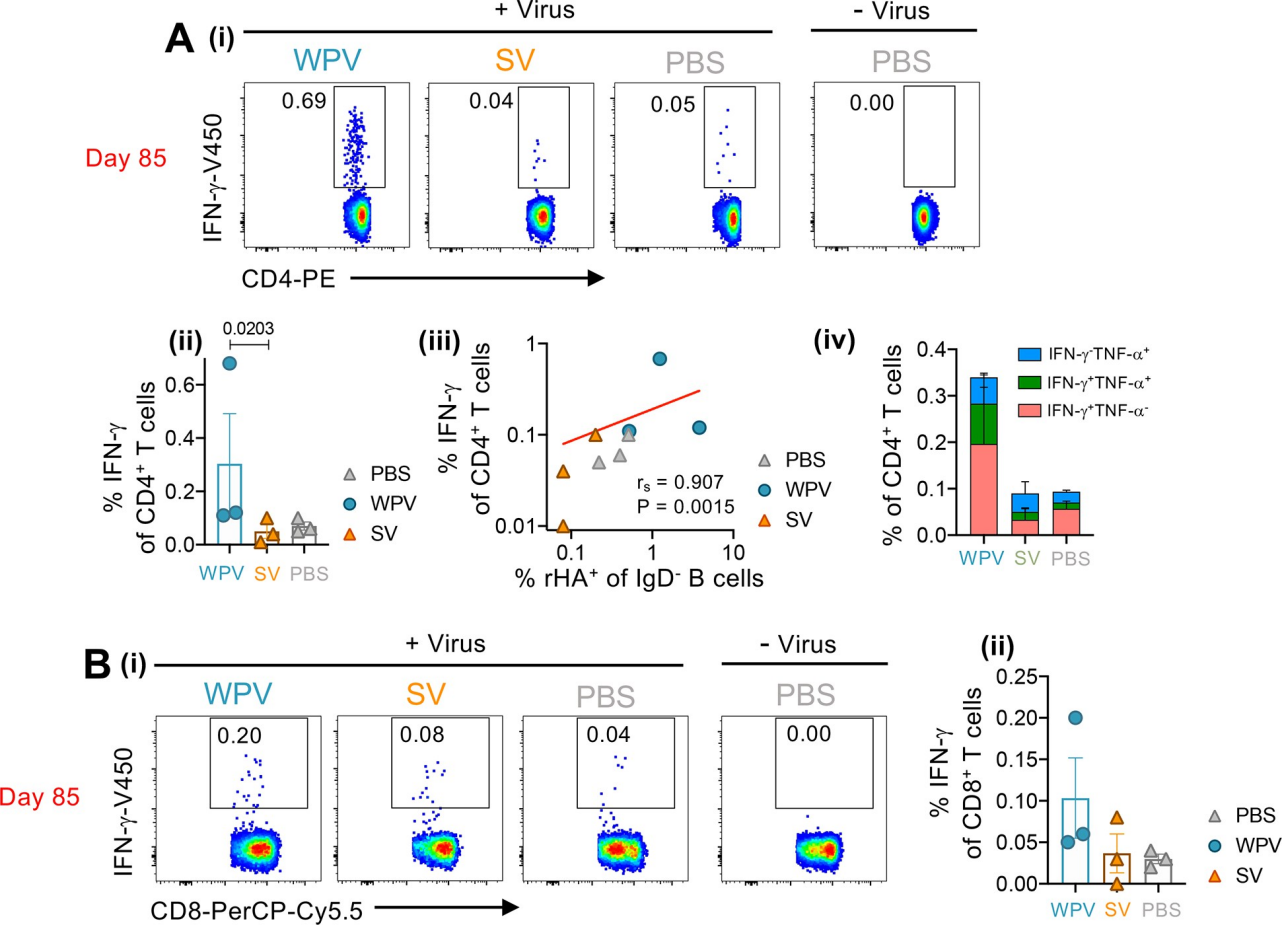
Higher frequencies of cytokine producing CD4^+^ T cells are detected in WPV vaccinated macaques. Representative dot plots of IFN-γ-producing (A[i]) CD4^+^ and (B[i]) CD8^+^ T cells detected in PBMCs samples following *in vitro* virus stimulation and their frequencies (A[ii] and B[ii]) within each group (mean±SD). (A[iii]) Correlation of IFN-γ-producing CD4^+^ T cells and frequencies (%) of influenza-virus specific B cells in individual animals from all groups showing the Spearman’s ranked correlation coefficient (r_s_) and P-value. (A[iv]) Influenza virus-specific CD4+ T cells were further characterized by analysing TNF-α production. Bar graphs depict the frequencies of cells producing IFN-γ or TNF-α only, or both (mean±SEM). Analysis of data comparing all groups was assessed by Kruskal-Wallis tests.

We also investigated the effects of WPV and SV immunization on virus-specific T cell responses at 8 days after the secondary influenza virus infection (day 85) by performing intracellular cytokine staining of PBMCs following an overnight stimulation with live A/Singapore/GP1908/2015 [25, 29, 30]. IFN-γ production in CD4^+^ and CD8^+^ T cells (S5B Fig) was detected in virus- but not in mock-infected cultures (Fig 6A[i] and 6B[i]). In comparison to SV-vaccinated animals, higher frequencies of IFN-γ-producing T cells were detected in animals vaccinated with WPV (Fig 6A[ii] and 6B[ii]), with significant differences in levels of IFN-γ^+^CD4^+^ T cells (P = 0.0203). There was also a strong positive correlation between IFN-γ^+^CD4^+^ T cells with rHA^+^ B cell frequencies (r_s_ = 0.907, P = 0.0015) detected amongst all groups (Fig 6A[iii]), in line with their importance in promoting B cell responses. Analysis of CD4^+^ T cells producing both IFN-γ and TNF-α showed that some WPV-vaccinated animals had higher frequencies of these polyfunctional populations (Fig 6A[iv]), suggesting their potential to establish memory pools.

## Discussion

While SV formulations have been instrumental in reducing global disease burden caused by seasonal influenza viruses, their effectiveness, especially in immunologically naïve individuals, remains suboptimal. Amongst the many different formulations of influenza vaccines that have been evaluated, studies have indicated that the use of WPV can be of a great benefit [21–23]. This is based on the view that inactivated intact virions are more immunogenic and induce greater seroconversion and protection than ether- or detergent-disrupted or subunit-based vaccine approaches. Concerns of WPV reactogenicity [15, 16], characterised by adverse effects associated with excessive inflammatory responses, however, have hindered development of WPVs. In the present study, conducted under the auspices of the All-Japan Influenza Vaccine Study Group with all four of Japan’s seasonal influenza vaccine manufacturers, highly pure virus preparations were used to avoid reactogenicity. In line with our previous findings in mice [31], acute systemic cytokine profiles and clinical symptoms of cynomolgus macaques vaccinated with WPV were equivalent to those elicited by their SV counterparts and yet still exhibited greater immunogenicity.

While methods to analyse influenza-specific immune responses in mice and humans are well established, similar techniques have been less developed for non-human primates. Here, we have applied an array of techniques and use of cross-reactive reagents to comprehensively probe influenza-specific responses in vaccinated cynomolgus macaques. In immunologically-naïve settings, stronger humoral responses were observed in WPV-immunized animals. The multiplex array used here to simultaneously measure 28 antibody features revealed enhanced antibody responses induced by monovalent and quadrivalent WPVs, directed against a broad range of representative viral antigens expressed by A/H1N1, influenza B and to a lesser extent A/H3N2. As the multiplex array uses plasma at a specific dilution, an important consideration is that the volume used is sufficient for capturing differences in responses across all antigens examined. In the case of NA, this has resulted in a high baseline level but nonetheless still provided a sufficient window to detect the differences. The most prominent augmentation in antibody responses were linked to IgG antibodies directed against the HA of A/H1N1 and influenza B, and to some degree, the HA Stem of A/H1N1. Not surprisingly, in the case of HA-specific IgG, HA inhibition of each strain strongly correlated with these antibody responses, indicating their potential to inhibit viral entry. Given that antibodies directed to the conserved HA Stem region are noted for their ability to cross-react with different HA subtypes within the same antigenic group [32], our data suggest that vaccination with WPV may also confer some level of heterosubtypic immunity against group 1 influenza virus strains. Of note, we did not observe significant increases in overall NA-specific antibody responses which could reflect the immunodominance of the HA or that NA amounts in these formulations are less abundant. Moreover, whilst we observed similarly high NP antibody responses induced by both vaccine types, these did not appear to be associated with HI antibody titres and protective potential [33].

In addition to binding via the antigen-binding fragment (Fab) region of the antibodies, the crystallizable fragment (Fc) region of antibodies plays a key role in recruitment of complement and activation of cellular effector mechanisms, most notably via ADCP by FcγR2a [34] and ADCC by FcγR3a [35–37]. Increased interactions of WPV-induced antigen-specific antibodies with these receptors suggests that apart from direct inhibition of viral entry in the event of infection, these antibodies also have the potential to engage Fc receptors on effector cells to facilitate cytolysis of virally-infected cells [38] and phagocytosis of opsonized material by cells expressing these receptors [34], thus can contribute to overall protection against infection and disease severity [39]. The engineering of low affinity FcγR ectodomains as a linked dimer confers avid binding only by pairs of IgG that occupy closely spaced (“near-neighbor”) epitopes [40]. The increased binding of dimeric FcγR to HA and HA Stem antibodies elicited by WPVs over those elicited by the equivalent SV vaccinations indicates a higher antibody response against these regions. FcγR-mediated antiviral activity, in addition to direct roles in ADCC and ADCP, also includes antigen presentation by FcγR2a-expressing dendritic cells, which with FcγR2a augmented antibodies can induce potent protective cellular responses [41].

In the context of infection induced immunity, higher titers of HI antibodies were induced by WPV vaccination and further boosted following a secondary influenza virus infection. This was associated with increased IgG HA and HA Stem-specific antibodies, virus-specific CD4^+^ T cell responses and frequencies of HA-specific B cells, a higher proportion of which expressed IgG, suggesting a more mature response and antibodies which could have better binding affinities and/or avidities. An important point to note is that differences in vaccine-induced antibody responses between groups at later time points were observed by ELISA and not in the multiplex array. This could be because the antibody levels were relatively high and beyond the detection limits of the array. Given the disrupted nature of the antigens in SV compared to intact antigens in WPV, there could be additional differences in the epitopes bound by these antibodies, which we are unable to detect as both the ELISA and array measures responses against whole antigens. A further note to consider is that the antigens used in the ELISA and array are not identical to those in the vaccines and using better matched antigens could reveal more specific differences.

The enhanced immunogenicity of WPV formulations is likely attributed to the presence of viral RNA, providing stimulation of innate immune responses via TLR7 recognition and signaling [19, 42]. Although SV preparations also contain RNA, ether-disruption and other manufacturing processes are likely to lead to its degradation [19, 43] as opposed to being retained and protected within a virion of a WPV [19]. As particulates, these preparations may lend themselves to be endocytosed more efficiently by antigen-presenting cells and concurrently induce their activation [44, 45]. Moreover, the density of antigens expressed on a virion surface is also likely to be higher, in comparison to the disrupted antigenic components contained within the SV formulation, and conducive to promoting cross-linking of B cell receptors (BCRs). Although we could not phenotype CD4^+^ T cell populations, it is reasonable to speculate that follicular helper T cells also contribute to the enhanced responses observed by providing the necessary signals required, and/or acting synergistically with BCR cross-linking, to drive germinal center and plasmablast responses [46]. Notably, studies in mice have shown that WPV but not SV elicit early T cell-independent responses through B-cell intrinsic TLR7 signaling by RNA contained within, resulting in the production of higher-affinity antibodies [47].

Overall, our results support other studies which show that WPV formulations are immunogenic [19, 22, 24] and even with reduced reactogenicity, can confer significant improvements in immune responses, compared to what would otherwise be suboptimal/ineffective responses induced by SV. Key to our findings is the use of techniques to comprehensively dissect and discern differences in influenza-specific immune responses in a non-human primate model, even with the limited sample sizes in these groups. These results support the implementation of vaccination regimens using WPV for immunologically naïve individuals, such as the young as they offer the greatest benefit, in terms of immunogenicity, over conventional SV formulations. Our findings also support their use in individuals that have been previously infected or vaccinated. As the production of these vaccines do not require ether disruption, they can be fast-tracked with minimal impact to existing inactivated influenza vaccine manufacturing protocols. Monovalent formulations could be ideal for rapid deployment in the event of an emergent pandemic influenza virus while quadrivalent formulations would be suitable for seasonal implementation.

## Methods

### Ethics statement

All animal experiments were approved by the Institutional Animal Care and Use Committee (Approved ethics number IACUC435-001 and IACUC435-002) and was performed in accordance with the animal welfare by laws of SNBL, which is accredited by American Association for Accreditation of Laboratory Animal Care (AAALAC) International. Healthy female and male cynomolgus monkeys (*Macaca fascicularis*) weighing 2 to 3.5 kg and aged 2 to 3 years old, were purpose-bred in Cambodia and imported and maintained at Shin Nippon Biomedical Laboratories, Ltd.

### Viruses

The influenza virus strains A/Singapore/GP1908/2015(IVR-180) (H1N1), A/Singapore/INFIMH-16-0019/2016(IVR-186) (H3N2), B/Phuket/3073/2013 (Yamagata linage) and B/Maryland/15/2016 (NYMC BX-69A) (Victoria lineage), were provided by the National Institute of Infectious Diseases (NIID) in Japan. A/Yokohama/91/2007 (H1N1) was provided by the Yokohama City Institute of Public Health. Viruses were propagated in 10-day old embryonated chicken eggs or in MDCK cells. Collected allantoic fluids or culture supernatants were stored at -80°C until use.

### Vaccines

WPVs and SVs used in this study were manufactured and provided by KM Biologics Co. Ltd. (Japan). Monovalent formulations were produced from A/Singapore/GP1908/2015(IVR-180) (H1N1). Quadrivalent formulations were produced with strains used in 2017–2018 seasonal vaccines in Japan; A/Singapore/GP1908/2015(IVR-180) (H1N1), A/Singapore/INFIMH-16-0019/2016 (IVR-186) (H3N2), B/Phuket/3073/2013 (Yamagata linage), and B/Maryland/15/2016 (NYMC BX-69A) (Victoria lineage). WPVs were prepared from highly purified virions and inactivated with formalin and/or β-propiolactone according to standard methods used by the manufacturer. SVs were prepared by disrupting purified virions with ether in accordance with manufacturing licenses and protocols for producing seasonal vaccines. HA protein concentrations of WPV and SV were quantified using the single-radial-immunodiffusion method.

### Animals and vaccination

All vaccine doses were administered at amounts equivalent to 15μg protein of the HA. For prime and boost experiments comparing monovalent formulations (Fig 1A), macaques were vaccinated with WPV or SV via the subcutaneous route on day 0 and 28. Blood samples were collected prior to each vaccine dose on day 0 and 28 as well as 6 and 24 hours after the first dose. In addition, a follow up bleed was collected on day 56. For experiments comparing quadrivalent formulations (Fig 3A), macaques were vaccinated with WPV or SV and bled at similar timepoints prior to each dose in addition to 6 and 24 hours after each vaccine was administered, and on day 49.

For experiments examining how previous influenza virus infection affects vaccine-induced responses (Fig 5A), macaques were first intranasally infected with 4×10^5^ TCID_50_/ml (in a volume of 1ml) of pre-pandemic strain of influenza A/Yokohama/91/2007 (H1N1) on day 0 followed by vaccination with monovalent WPV or SV or PBS on day 56. All macaques were then challenged with 2×10^5^ TCID_50_/ml (in a volume of 2ml) of the 2009 pandemic stain A/Narita/1/2009 (H1N1) on day 77. Blood samples were collected on day 0, 56 and 77 prior to each intervention as well as on day 85. All blood samples were collected in heparin tubes (BD Biosciences, Franklin Lakes, NJ, USA) from the femoral vein and plasma and peripheral blood mononuclear cells (PBMCs) separated and stored at -80°C until use. Rectal temperatures were measured in non-anesthetized animals using a C402 or C405 digital thermometer (Terumo, Japan).

### Hemagglutination-inhibition (HI) assay

Plasma were treated with receptor-destroying enzyme (RDE; Denka Seiken, Tokyo, Japan) at a plasma:RDE ratio of 1:3 and incubated for 16–18 hours at 37°C and then at 56°C for 1 hour. RDE-treated plasma were serially diluted two-fold with PBS in 96-well microplates. The diluted plasma were mixed with 8 hemagglutinin units of virus antigen and incubated at room temperature for 30 min. Chicken red blood cells (0.5%) were added to the antigen-plasma dilution mixtures and incubated at room temperature for a further 30 min. HI titres were expressed as reciprocals of the highest plasma dilutions that showed complete hemagglutination.

### Infection of PBMCs with influenza viruses and intracellular cytokine staining

Stimulation of macaque PBMCs with live influenza virus was performed as previously described [25]. Cryopreserved PBMCs were thawed at 37°C in serum-free RPMI 1640 medium (Thermo Fisher Scientific, MA, USA) supplemented with 10% heat-inactivated fetal bovine serum (FBS), 1 mM of sodium pyruvate (Thermo Fisher Scientific), 50 μM of 2-mercaptoethanol (Merck, Darmstadt, Germany), 100 U/ml of penicillin (Thermo Fisher Scientific), 100 μg/ml of streptomycin (Thermo Fisher Scientific), and 20 μg/ml of gentamicin (Thermo Fisher Scientific). Approximately 10^6^ PBMC were infected with live influenza A virus at a multiplicity of infection (MOI) of 6 or with media alone in serum-free media at 37°C for 1 hour. FBS was added (final concentration of 10%) and cells incubated for a further 3 hours. Golgi Plug (BD Biosciences, USA) was then introduced at a final concentration of 1 μg/ml to inhibit protein export and cells incubated for a further 16 hours. Cells were harvested and stained with surface antibodies (S3 Table) in 50 μl of stain buffer (1% FBS–5mM EDTA–PBS) for 30 mins at 4°C. Following washing with fresh stain buffer, cells were fixed and permeabilized in 100 μl CytoFix/CytoPerm (BD Biosciences) for 20 mins and then stained with antibodies to detect intracellular cytokines (S3 Table) in 50μl of Perm/Wash buffer (BD Biosciences) for 30 mins at 4°C. Samples were acquired using a BD LSR Fortessa (BD Biosciences, USA) and data analysed by FlowJo software (BD Biosciences, USA).

### Recombinant HA staining of PBMCs

Recombinant HA (rHA) probes specific for the A/California/07/2009(H1N1)pdm09 HA were generated and used for staining HA-specific B cells as described [25, 48, 49]. Thawed PBMCs were stained with antibodies and rHA probes (S4 Table) at 4°C for 30 mins in PBS containing 1% FBS and fixed in 1% paraformaldehyde prior to analysis by flow cytometry. B cells were identified as CD45^+^CD19^+^CD20^+^ cells within live events (S5A Fig). Influenza virus-specific B cells were identified as rHA^+^ within class-switched (IgD^-^) B cells.

### Cytokine measurement by cytometric bead array

Cytokine levels in plasma samples from vaccinated macaques were analyzed using a BD Human Cytometric Bead Array kit according to the manufacturer’s instructions and as previously described [25].

### Multiplex array

Antigens were covalently coupled to magnetic carboxylated beads using a two-step carbodiimide reaction as previously described [26, 27]. Briefly, antigen-coupled beads (500–750 beads per bead region) were pooled and combined with diluted plasma (IgG; 1:600, IgA; 1:400, FcγR2 and FcγR3; 1:200) overnight before washing and staining with detectors (PE-conjugated anti-human IgG and IgA antibodies or soluble dimeric FcγR followed by streptavidin PE conjugate) (S2 Table). Plates were washed and read by a FlexMap 3D System (Luminex), with the binding of the PE detectors measured to calculate the median fluorescence intensity (MFI).

### Enzyme-linked immunosorbent assays (ELISAs)

Antigens (2μg/ml PBS) were coated onto Nunc MaxiSorp flat bottom 96-well plates (Thermo Fisher Scientific) for a minimum of 24 hours. Removal of antigen was followed by blocking of wells using PBS (containing w/v 10% BSA) for 1 hour. Plasma was serially diluted across each plate in PBS (containing v/v 0.05% Tween and w/v 5% BSA) and incubated for 2 hours at room temperature. For the detection of IgG, wells were incubated with alkaline phosphate-conjugated mouse anti-human IgG (MT78, MabTech) at a 1:1000 dilution for 1 hour and developed with pNPP substrate (Sigma) for 2 hours. Absorbance of wells were determined at 405nm on a Multiskan plate reader (Labsystems). All wells were washed extensively between steps using PBS (containing 0.05% Tween). Endpoint titers were determined by interpolation from a sigmodial curve fit (all R-squared values >0.95; GraphPad Prism 8) and expressed as the reciprocal of the highest dilution of plasma required to produce an absorbance value of 0.4.

### Statistical analysis

Prism 9 (GraphPad Software, San Diego, CA, USA) was used to perform statistical analyses. Where appropriate, data normality was tested using a Shapiro-Wilk test to inform the statistical analysis. For non-parametric analysis between 2 groups, an unpaired two-tailed Mann-Whitney test was used; for analysis between 3 or more groups, the Kruskal-Wallis test or two-way ANOVA with Tukey’s correction for multiple comparisons was used. A P value less than 0.05 was considered statistically significant.

## Supporting information

S1 Fig Food intake, body weight and temperature following vaccination with monovalent formulations. Macaques (n = 9 per group) were vaccinated with monovalent A/Singapore/GP1908/2015 (H1N1) WPV or SV followed by a second dose 28 days later. Animals were monitored for (A) amount of food consumed per day as well as (B) body weight and (C) temperature throughout the course of the experiment.
(TIFF)

S2 Fig Monovalent WPV induces stronger antibody responses than SV after one dose. Multiplex antibody responses (mean±SD) from (A) Fig 1D and (B) Fig 3C, against A/H1N1 and IBV antigens prior to vaccination (day 0), after the first (day 28) and second dose (day 49 or 56). Comparisons of responses between each time-point within each vaccine group was assessed using Kruskal-Wallis tests.
(TIFF)

S3 Fig WPV induces stronger antibody responses than SV by ELISA. (A) Plasma from animals vaccinated with monovalent A/Singapore/GP1908/2015 (H1N1) WPV or SV (day 28) followed by a second dose 28 days later (day 56) in Fig 1 were analysed for IgG antibody titres against HA (H1N1) and HA Stem (H1N1) by ELISA. Mann-Whitney tests were used to analyse statistical significance between vaccine groups with the mean±SD in each group shown. (B) Plasma from animals obtained on day 77 and 85 in Fig 5 were analysed for IgG antibody titres against HA (H1N1), HA Stem (H1N1) and NP (H1N1) by ELISA. Data comparing all groups and time points with each other was determined by two-way ANOVA.
(TIFF)

S4 Fig Quadrivalent WPV induces stronger antibody responses and HI antibody titres to vaccine matched strains than SV. (A) Multiplex antibody responses (mean±SD) from Fig 3C and (B) HI antibody titres (mean±SD) from Fig 4A, against A/H1N1, A/H3N2 and IBV antigens on day 49, with data represented as separate groups and analysis by Kruskal-Wallis tests. Asterisks indicate P-values of <0.05.
(TIFF)

S5 Fig Gating strategy used to define PBMC populations. (A) Detection of A/H1N1 virus-specific B cells in PBMCs were analysed by gating CD19^+^ CD20^+^class switched (IgD^-^) rHA^+^cells and could be further defined by IgM or IgG expression. (B) CD8^+^ and CD4^+^ expressing PBMCs were gated for CD3 expression to define IFN-γ and/or TNF-α cytokine producing CD3^+^CD8^+^ and CD3^+^CD4^+^ T cells.
(TIFF)

S1 Data Source data file. Raw data for all the figures and supplementary figures.
(XLSX)

S1 Table Clinical symptoms and injection site observations following vaccination. (DOCX)

S2 Table Panel design of the multiplex bead array assay. (DOCX)

S3 Table Flow cytometry panel for the analysis of influenza-specific T cells. (DOCX)

S4 Table Flow cytometry panel for the analysis of influenza-specific B cells. (DOCX)

S5 Table Viral titers in nasal swabs of animals following A/Narita/1/2009 infection. (DOCX)
