# Supplementary figures and images for: Immunization with inactivated whole virus particle influenza virus vaccines improves the humoral response landscape in cynomolgus macaques
Source: PLoS Pathog. 2022 Oct 7;18(10):e1010891. doi: 10.1371/journal.ppat.1010891 (PMC9581423; doi:10.1371/journal.ppat.1010891)

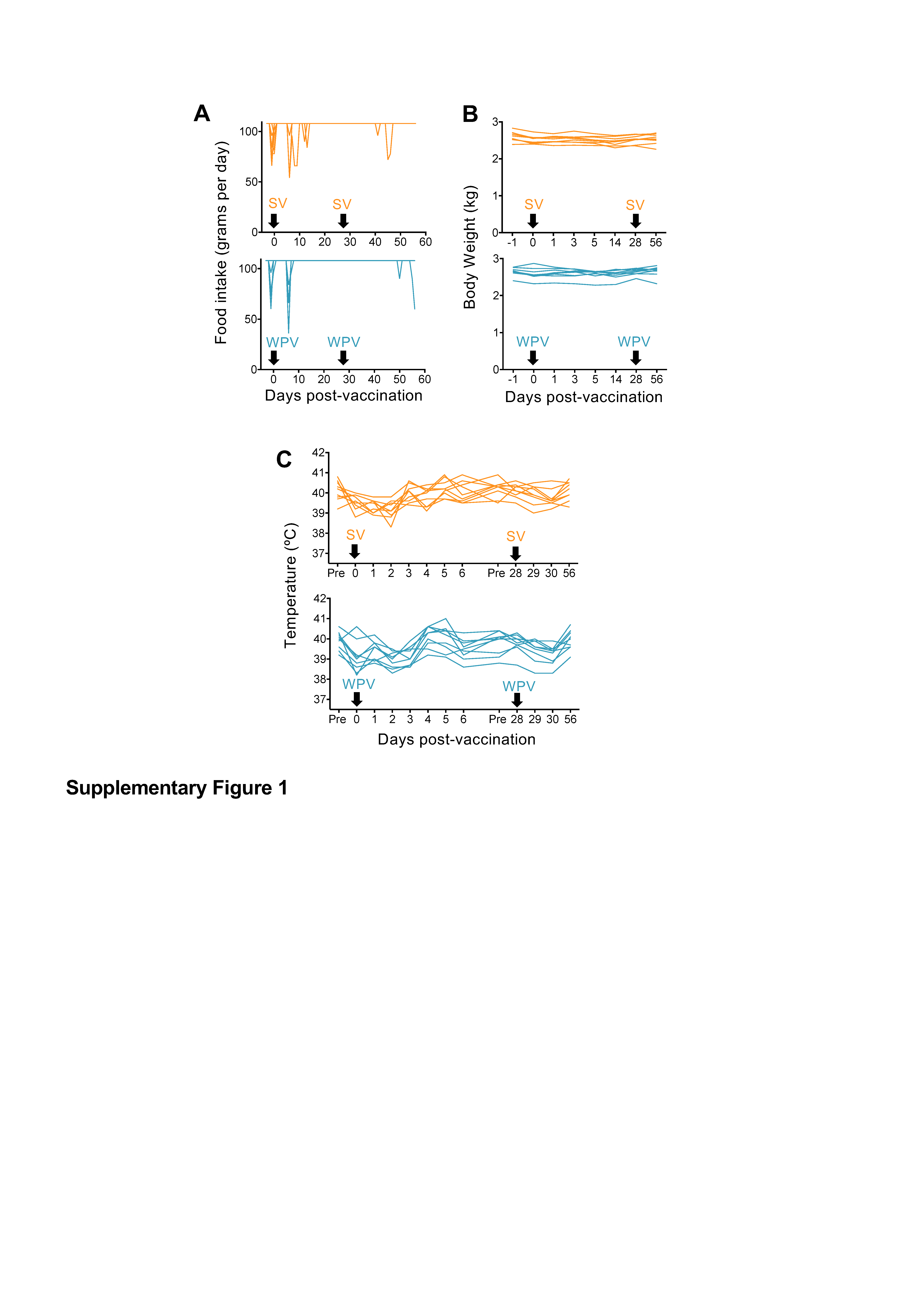

Supplement: S1 Fig — Macaques (n = 9 per group) were vaccinated with monovalent A/Singapore/GP1908/2015 (H1N1) WPV or SV followed by a second dose 28 days later. Animals were monitored for (A) amount of food consumed per day as well as (B) body weight and (C) temperature throughout the course of the experiment. (TIFF) [file ppat.1010891.s001.tiff]

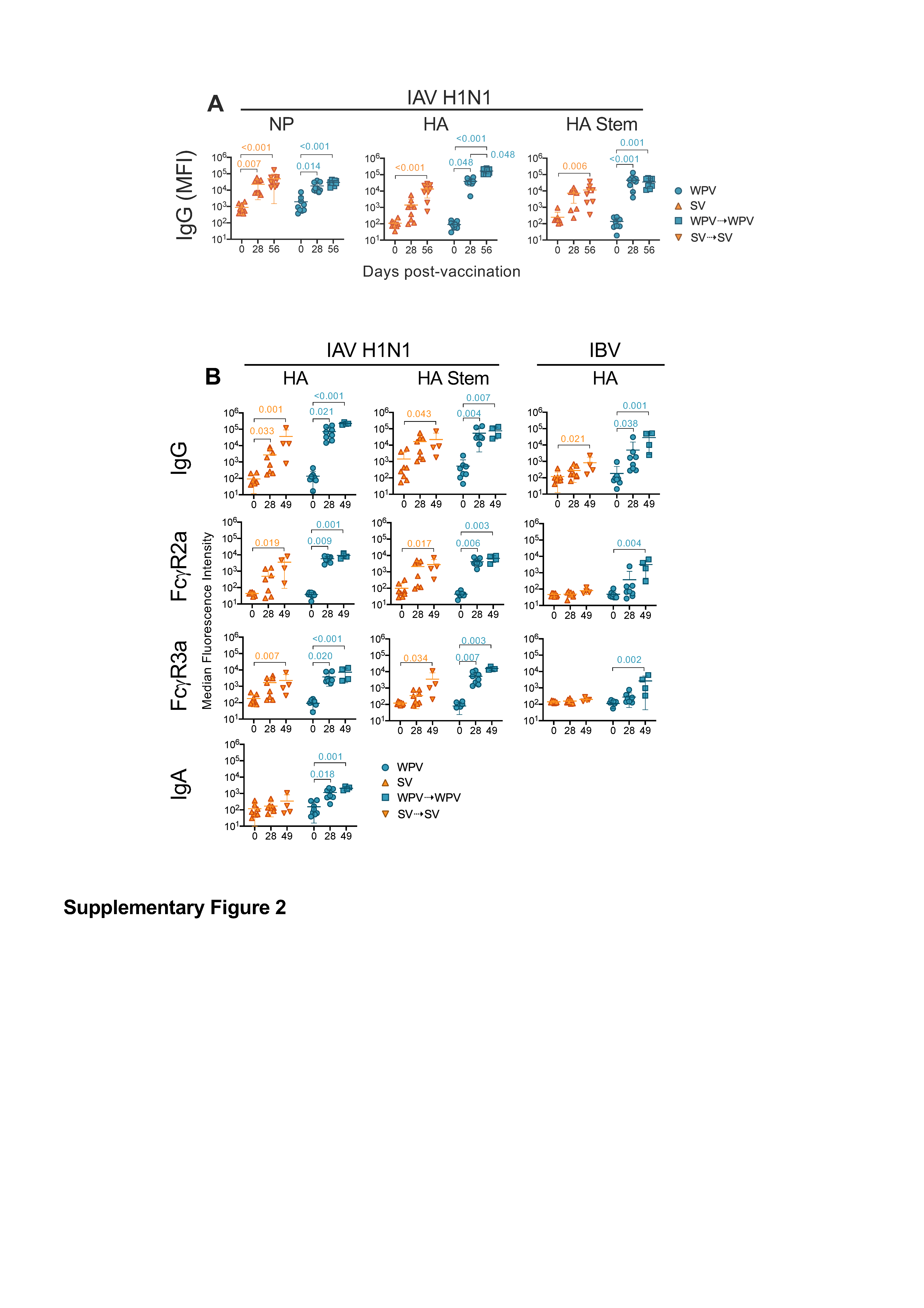

Supplement: S2 Fig — Multiplex antibody responses (mean±SD) from (A) Fig 1D and (B) Fig 3C, against A/H1N1 and IBV antigens prior to vaccination (day 0), after the first (day 28) and second dose (day 49 or 56). Comparisons of responses between each time-point within each vaccine group was assessed using Kruskal-Wallis tests. (TIFF) [file ppat.1010891.s002.tiff]

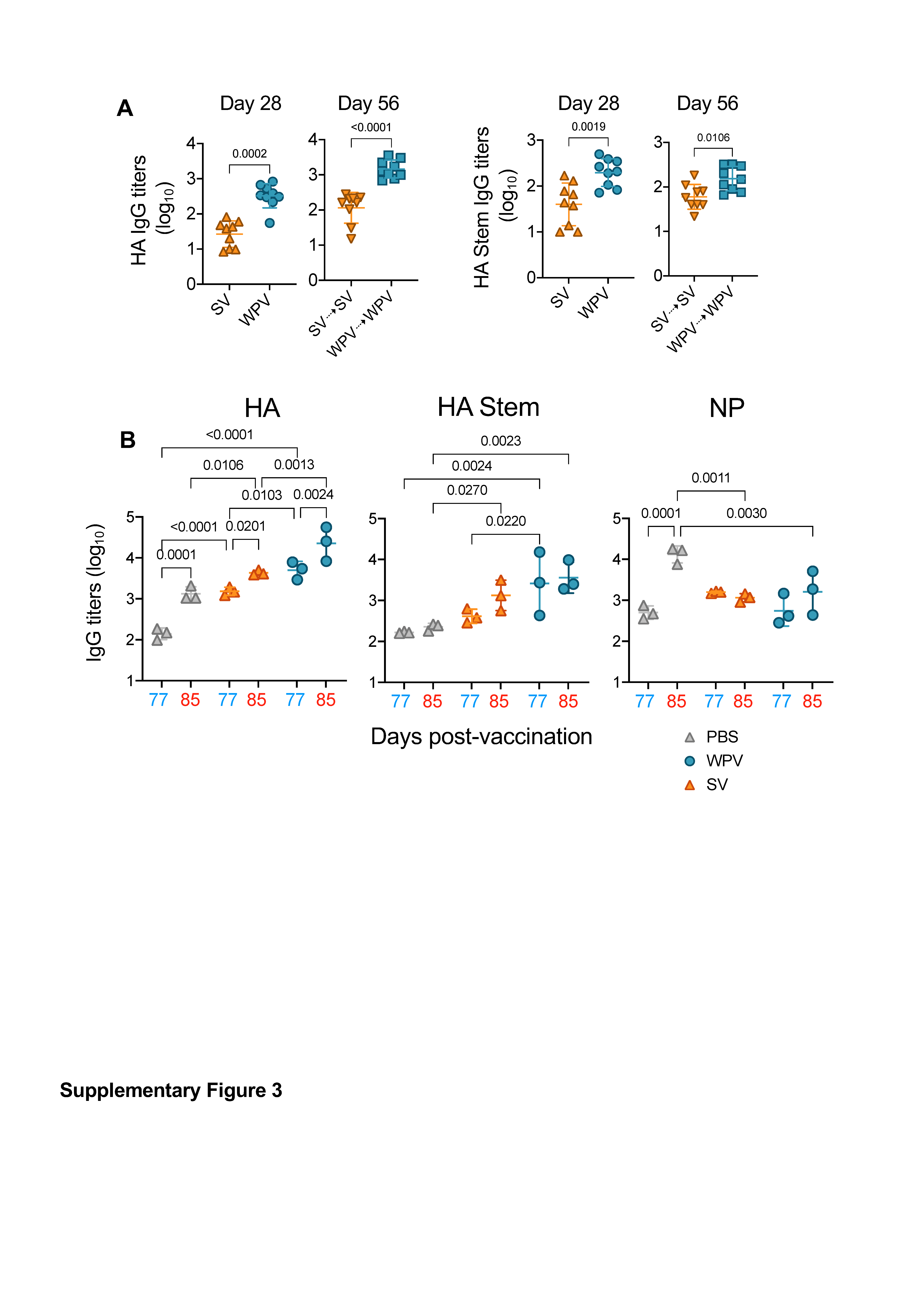

Supplement: S3 Fig — (A) Plasma from animals vaccinated with monovalent A/Singapore/GP1908/2015 (H1N1) WPV or SV (day 28) followed by a second dose 28 days later (day 56) in Fig 1 were analysed for IgG antibody titres against HA (H1N1) and HA Stem (H1N1) by ELISA. Mann-Whitney tests were used to analyse statistical significance between vaccine groups with the mean±SD in each group shown. (B) Plasma from animals obtained on day 77 and 85 in Fig 5 were analysed for IgG antibody titres against HA (H1N1), HA Stem (H1N1) and NP (H1N1) by ELISA. Data comparing all groups and time points with each other was determined by two-way ANOVA. (TIFF) [file ppat.1010891.s003.tiff]

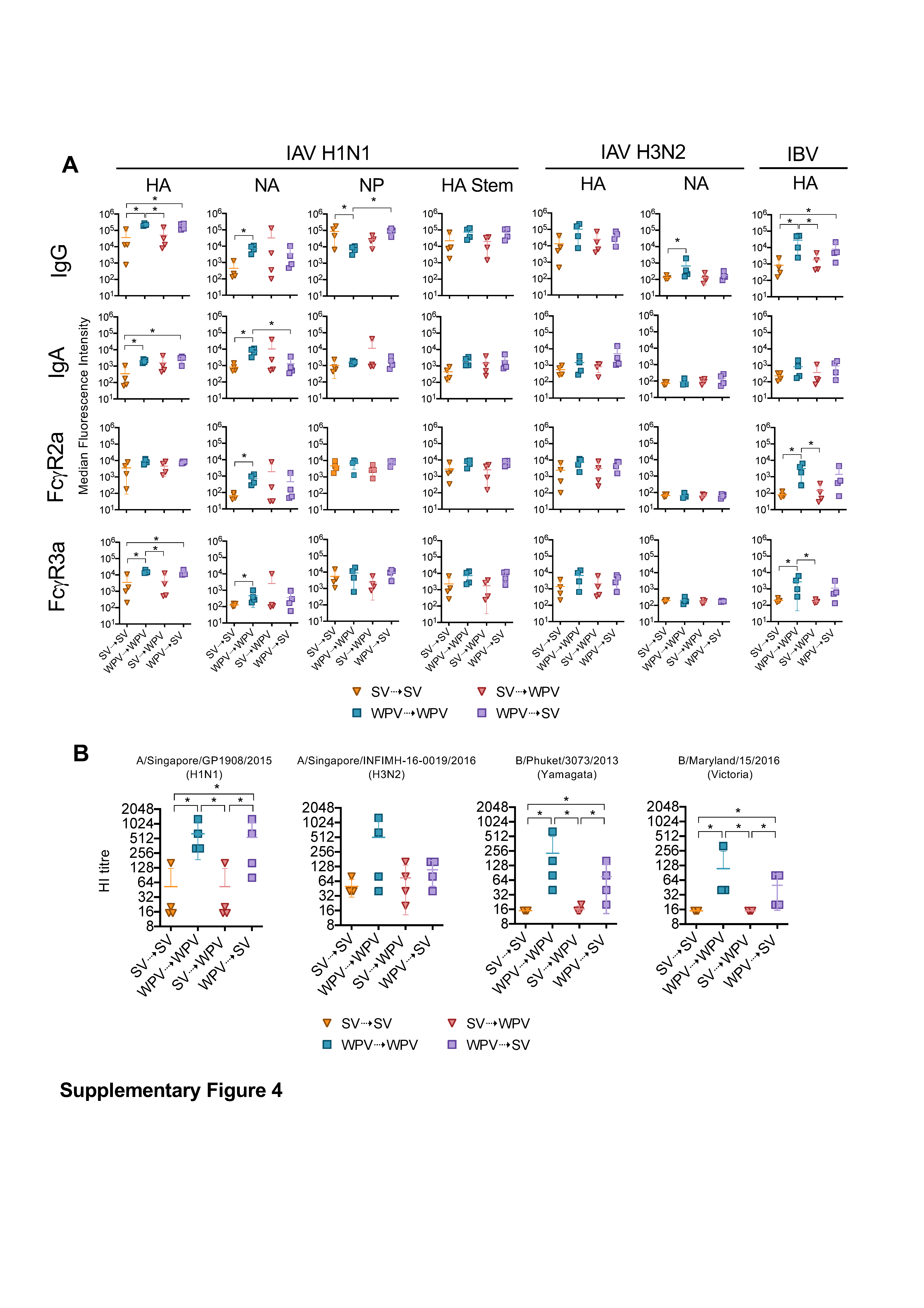

Supplement: S4 Fig — (A) Multiplex antibody responses (mean±SD) from Fig 3C and (B) HI antibody titres (mean±SD) from Fig 4A, against A/H1N1, A/H3N2 and IBV antigens on day 49, with data represented as separate groups and analysis by Kruskal-Wallis tests. Asterisks indicate P-values of <0.05. (TIFF) [file ppat.1010891.s004.tiff]

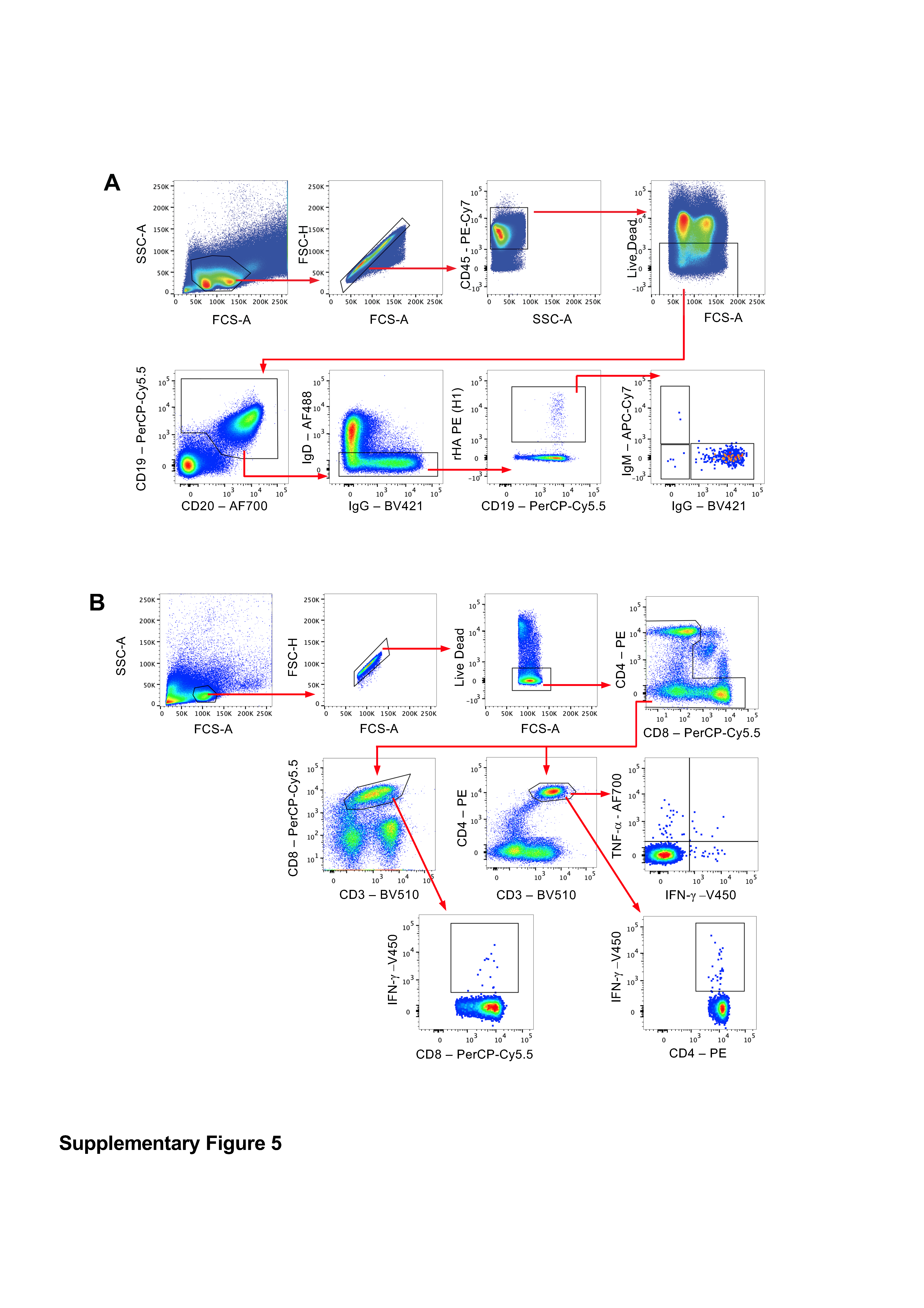

Supplement: S5 Fig — (A) Detection of A/H1N1 virus-specific B cells in PBMCs were analysed by gating CD19+ CD20+class switched (IgD-) rHA+cells and could be further defined by IgM or IgG expression. (B) CD8+ and CD4+ expressing PBMCs were gated for CD3 expression to define IFN-γ and/or TNF-α cytokine producing CD3+CD8+ and CD3+CD4+ T cells. (TIFF) [file ppat.1010891.s005.tiff]
