# Supplementary material for: Immunization with inactivated whole virus particle influenza virus vaccines improves the humoral response landscape in cynomolgus macaques
Source: PLoS Pathog. 2022 Oct 7;18(10):e1010891. doi: 10.1371/journal.ppat.1010891 (PMC9581423; doi:10.1371/journal.ppat.1010891)
Supplement: S1 Table — (DOCX) [file ppat.1010891.s007.docx]

**Supplementary Table 1.**

Clinical symptoms and injection site observations following vaccination

|  | | | | Days after 1^st^ dose | | | | | | | | | | | | | | | | | | | | | | | | | | | |
| --- | --- | --- | --- | --- | --- | --- | --- | --- | --- | --- | --- | --- | --- | --- | --- | --- | --- | --- | --- | --- | --- | --- | --- | --- | --- | --- | --- | --- | --- | --- | --- |
| Vaccine | Number | Gender | Sign(s) | 1 | 2 | 3 | 4 | 5 | 6 | 7 | 8 | 9 | 10 | 11 | 12 | 13 | 14 | 15 | 16 | 17 | 18 | 19 | 20 | 21 | 22 | 23 | 24 | 25 | 26 | 27 | 28 |
| WPV | 1 | Male |  | - | - | - | - | - | - | - | - | - | - | - | - | - | - | - | - | - | - | - | - | - | - | - | - | - | - | - | - |
|  | 2 | Male |  | - | - | - | - | - | - | - | - | - | - | - | - | - | - | - | - | - | - | - | - | - | - | - | - | - | - | - | - |
|  | 3 | Female |  | - | - | - | - | - | - | - | - | - | - | - | - | - | - | - | - | - | - | - | - | - | - | - | - | - | - | - | - |
|  | 4 | Male |  | - | - | - | - | - | - | - | - | - | - | - | - | - | - | - | - | - | - | - | - | - | - | - | - | - | - | - | - |
|  | 5 | Female |  | - | - | - | - | - | - | - | - | - | - | - | - | - | - | - | - | - | - | - | - | - | - | - | - | - | - | - | - |
|  | 6 | Female | S | - | - | - | - | - | - | - | - | - | - | 1 | 1 | 1 | - | - | - | - | - | - | - | - | - | - | - | - | - | - | - |
|  |  |  | T |  |  |  |  |  |  |  |  |  |  | + | + | + | + |  |  |  |  |  |  |  |  |  |  |  |  |  |  |
|  | 7 | Male |  | - | - | - | - | - | - | - | - | - | - | - | - | - | - | - | - | - | - | - | - | - | - | - | - | - | - | - | - |
|  | 8 | Female |  | - | - | - | - | - | - | - | - | - | - | - | - | - | - | - | - | - | - | - | - | - | - | - | - | - | - | - | - |
|  | 9 | Female |  | - | - | - | - | - | - | - | - | - | - | - | - | - | - | - | - | - | - | - | - | - | - | - | - | - | - | - | - |
| SV | 10 | Male |  | - | - | - | - | - | - | - | - | - | - | - | - | - | - | - | - | - | - | - | - | - | - | - | - | - | - | - | - |
|  | 11 | Male |  | - | - | - | - | - | - | - | - | - | - | - | - | - | - | - | - | - | - | - | - | - | - | - | - | - | - | - | - |
|  | 12 | Female |  | - | - | - | - | - | - | - | - | - | - | - | - | - | - | - | - | - | - | - | - | - | - | - | - | - | - | - | - |
|  | 13 | Male |  | - | - | - | - | - | - | - | - | - | - | - | - | - | - | - | - | - | - | - | - | - | - | - | - | - | - | - | - |
|  | 14 | Female |  | - | - | - | - | - | - | - | - | - | - | - | - | - | - | - | - | - | - | - | - | - | - | - | - | - | - | - | - |
|  | 15 | Female |  | - | - | - | - | - | - | - | - | - | - | - | - | - | - | - | - | - | - | - | - | - | - | - | - | - | - | - | - |
|  | 16 | Male |  | - | - | - | - | - | - | - | - | - | - | - | - | - | - | - | - | - | - | - | - | - | - | - | - | - | - | - | - |
|  | 17 | Female |  | - | - | - | - | - | - | - | - | - | - | - | - | - | - | - | - | - | - | - | - | - | - | - | - | - | - | - | - |
|  | 18 | Female | S | - | - | - | - | - | - | - | - | 2 | 2 | 2 | 2 | 2 | 1 | - | - | - | - | - | - | - | - | - | - | - | - | - | - |
|  |  |  | C |  |  |  |  |  |  |  |  |  |  |  |  |  | + | + | + | + | + | + | + | + |  |  |  |  |  |  |  |
|  |  |  | T |  |  |  |  |  |  |  |  | + | + | + | + | + |  |  |  |  |  |  |  |  |  |  |  |  |  |  |  |

|  |  |  |  | Days after 1^st^ dose | | | | | | | | | | | | | | | | | | | | | | | | | | | |
| --- | --- | --- | --- | --- | --- | --- | --- | --- | --- | --- | --- | --- | --- | --- | --- | --- | --- | --- | --- | --- | --- | --- | --- | --- | --- | --- | --- | --- | --- | --- | --- |
| Vaccine | Number | Gender | Sign(s) | 29 | 30 | 31 | 32 | 33 | 34 | 35 | 36 | 37 | 38 | 39 | 40 | 41 | 42 | 43 | 44 | 45 | 46 | 47 | 48 | 49 | 50 | 51 | 52 | 53 | 54 | 55 | 56 |
| WPV⇢WPV | 1 | Male |  | - | - | - | - | - | - | - | - | - | - | - | - | - | - | - | - | - | - | - | - | - | - | - | - | - | - | - | - |
|  | 2 | Male |  | - | - | - | - | - | - | - | - | - | - | - | - | - | - | - | - | - | - | - | - | - | - | - | - | - | - | - | - |
|  | 3 | Female |  | - | - | - | - | - | - | - | - | - | - | - | - | - | - | - | - | - | - | - | - | - | - | - | - | - | - | - | - |
|  | 4 | Male |  | - | - | - | - | - | - | - | - | - | - | - | - | - | - | - | - | - | - | - | - | - | - | - | - | - | - | - | - |
|  | 5 | Female |  | - | - | - | - | - | - | - | - | - | - | - | - | - | - | - | - | - | - | - | - | - | - | - | - | - | - | - | - |
|  | 6 | Female |  | - | - | - | - | - | - | - | - | - | - | - | - | - | - | - | - | - | - | - | - | - | - | - | - | - | - | - | - |
|  | 7 | Male |  | - | - | - | - | - | - | - | - | - | - | - | - | - | - | - | - | - | - | - | - | - | - | - | - | - | - | - | - |
|  | 8 | Female |  | - | - | - | - | - | - | - | - | - | - | - | - | - | - | - | - | - | - | - | - | - | - | - | - | - | - | - | - |
|  | 9 | Female |  | - | - | - | - | - | - | - | - | - | - | - | - | - | - | - | - | - | - | - | - | - | - | - | - | - | - | - | - |
| SV⇢SV | 10 | Male |  | - | - | - | - | - | - | - | - | - | - | - | - | - | - | - | - | - | - | - | - | - | - | - | - | - | - | - | - |
|  | 11 | Male |  | - | - | - | - | - | - | - | - | - | - | - | - | - | - | - | - | - | - | - | - | - | - | - | - | - | - | - | - |
|  | 12 | Female | M | - | - | - | - | - | - | - | - | - | - | - | + | + | + | + | - | - | - | - | - | - | - | - | - | - | - | - | - |
|  | 13 | Male |  | - | - | - | - | - | - | - | - | - | - | - | - | - | - | - | - | - | - | - | - | - | - | - | - | - | - | - | - |
|  | 14 | Female |  | - | - | - | - | - | - | - | - | - | - | - | - | - | - | - | - | - | - | - | - | - | - | - | - | - | - | - | - |
|  | 15 | Female | M | - | - | - | - | - | - | - | - | - | - | - | + | + | + | + | - | - | - | - | - | - | - | - | - | - | - | - | - |
|  | 16 | Male |  | - | - | - | - | - | - | - | - | - | - | - | - | - | - | - | - | - | - | - | - | - | - | - | - | - | - | - | - |
|  | 17 | Female |  | - | - | - | - | - | - | - | - | - | - | - | - | - | - | - | - | - | - | - | - | - | - | - | - | - | - | - | - |
|  | 18 | Female | M | - | - | - | - | - | - | - | - | - | - | - | + | + | + | + | - | - | - | - | - | + | - | - | - | - | - | - | - |

Abbreviations:

No abnormal signs (-); Non-graded signs (+); where graded, 1: slight, 2: Moderate, 3: Severe

Menstruation (M); traumatic stress (T); swelling at injection site (S); focal crust at injection site (C)
