# Supplementary material for: Immunization with inactivated whole virus particle influenza virus vaccines improves the humoral response landscape in cynomolgus macaques
Source: PLoS Pathog. 2022 Oct 7;18(10):e1010891. doi: 10.1371/journal.ppat.1010891 (PMC9581423; doi:10.1371/journal.ppat.1010891)
Supplement: S2 Table — (DOCX) [file ppat.1010891.s008.docx]

**S2 Table.**

Panel design of the multiplex bead array assay

| Influenza Strain | Protein^a^ |  | Isotypes & FcγR bindings |
| --- | --- | --- | --- |
| A/California/04/2009 (A/H1N1) | Trimeric HA |  | IgG |
|  | Monomeric NA |  | IgA |
| A/California/07/2009 (A/H1N1) | NP |  | FcγR2a |
|  | HA Stem |  | FcγR3a |
| A/Hong Kong/4801/2014 (A/H3N2) | NA |  |  |
| A/Singapore/INFIMH-16-0019/2016 (A/H3N2) | HA |  |  |
| B/Phuket/3073/2013 (B/Yamagata) | HA |  |  |

^a^HA: hemagglutinin; NA: neuraminidase; NP: nucleoprotein
