# Supplementary material for: Immunization with inactivated whole virus particle influenza virus vaccines improves the humoral response landscape in cynomolgus macaques
Source: PLoS Pathog. 2022 Oct 7;18(10):e1010891. doi: 10.1371/journal.ppat.1010891 (PMC9581423; doi:10.1371/journal.ppat.1010891)
Supplement: S3 Table — (DOCX) [file ppat.1010891.s009.docx]

| **S3 Table.**  Flow cytometry panel for the analysis of influenza-specific T cells | | | | | |
| --- | --- | --- | --- | --- | --- |
| Specificity | Clone | Fluorochrome^a^ | Supplier^b^ | Dilution^c^ | Cat # |
| CD3 | SP34.2 | BV510 | BD | 1:150 | 740187 |
| CD4 | OKT4 | PE | Biolegend | 1:100 | 317410 |
| CD8 | SK1 | PerCP-Cy5.5 | Biolegend | 1:100 | 344710 |
| Live Dead | N/A | NIR | ThermoFisher | 1:500 | L34975 |
| IFN-γ | Β27 | V450 | BD | 1:100 | 560371 |
| TNF-α | MAB11 | AF700 | Biolegend | 1:50 | 502928 |
| ^a^Fluorochrome abbreviations: BV: Brilliant Violet; PE: phycoerythrin; PerCP: Peridin-chlorophyll; Cy5.5: Cyanine-5.5; NIR: Near infrared; AF: Alexa-Fluor  ^b^BD: BD Biosciences, California, USA; Biolegend: Biolegend, California, USA; ThermoFisher: ThermoFisher Scientific, Massachusetts, USA; GeneTex: GeneTeX Inc, California, USA  ^c^Final staining volume is 50μl | | | | | |
