# Supplementary material for: Immunization with inactivated whole virus particle influenza virus vaccines improves the humoral response landscape in cynomolgus macaques
Source: PLoS Pathog. 2022 Oct 7;18(10):e1010891. doi: 10.1371/journal.ppat.1010891 (PMC9581423; doi:10.1371/journal.ppat.1010891)
Supplement: S4 Table — (DOCX) [file ppat.1010891.s010.docx]

| **S4 Table.**  Flow cytometry panel for the analysis of influenza-specific B cells | | | | | |
| --- | --- | --- | --- | --- | --- |
| Specificity | Clone | Fluorochrome^b^ | Supplier^c^ | Dilution^d^ | Cat # |
| CD45 | D058-1283 | PE-Cy7 | BD | 1:300 | 561294 |
| CD19 | J3-119 | PerCP-Cy5.5 | BC | 1:30 | A66328 |
| CD20 | 2H7 | AF700 | Biolegend | 1:150 | 302322 |
| IgD | Polyclonal | AF488 | SB | 1:150 | 3020-30 |
| IgG | G18-145 | BV421 | BD | 1:100 | 562581 |
| IgM | MHM-88 | APC-Cy7 | Biolegend | 1:100 | 314520 |
| rHA^a^ | N/A | PE | in-house^e^ | 5ul/sample | N/A |
| Live Dead | N/A | Aqua | ThermoFisher | 1:500 | L34965 |
| ^a^rHA: recombinant hemagglutinin  ^b^Fluorochrome abbreviations: PE: phycoerythrin; Cy7: Cyanine-7; PerCP: Peridin-chlorophyll; Cy5.5: Cyanine-5.5; AF: Alexa-Fluor; BV: Brilliant Violet; APC: allophycocyanin  ^c^BD: BD Biosciences, California, USA; BC: Beckman Coulter, California, USA; SB: SouthernBiotech, Alabama, USA; Biolegend: Biolegend, California, USA; ThermoFisher: ThermoFisher Scientific, Massachusetts, USA  ^d^Final staining volume is 150μl  ^e^See references 44 and 45 for details | | | | | |
