# Supplementary material for: Immunization with inactivated whole virus particle influenza virus vaccines improves the humoral response landscape in cynomolgus macaques
Source: PLoS Pathog. 2022 Oct 7;18(10):e1010891. doi: 10.1371/journal.ppat.1010891 (PMC9581423; doi:10.1371/journal.ppat.1010891)
Supplement: S5 Table — (DOCX) [file ppat.1010891.s011.docx]

**S5 Table.**

Viral titers in nasal swabs of animals following A/Narita/1/2009 infection

|  |  | Days after infection^a^ | | |
| --- | --- | --- | --- | --- |
| Group | Animal | 1 | 3 | 5 |
| PBS | 1 | 2.67 | 4.50 | 2.33 |
|  | 2 | 3.00 | 3.67 | 1.50 |
|  | 3 | 2.50 | 3.83 | ≦0.67 |
| SV | 1 | - | - | - |
|  | 2 | - | - | - |
|  | 3 | - | - | - |
| WPV | 1 | - | ≦0.67 | - |
|  | 2 | - | - | - |
|  | 3 | - | - | - |

^a^Viral titers expressed as log_10_ PFU/ml as determined by MDCK plaque assays

Dashes indicate undetectable viral plaques
